# Eco‐Evolutionary Optimality in Soil Organic Matter Models

**DOI:** 10.1111/ele.70278

**Published:** 2025-12-19

**Authors:** Erik Schwarz, Elsa Abs, Arjun Chakrawal, Luciana Chavez Rodriguez, Pierre Quévreux, Stefano Manzoni

**Affiliations:** ^1^ Department of Physical Geography Stockholm University Stockholm Sweden; ^2^ Bolin Centre for Climate Research Stockholm University Stockholm Sweden; ^3^ Laboratoire des Sciences du Climat et de l'Environnement Saint‐Aubin France; ^4^ Environmental Molecular Sciences Laboratory (EMSL), Pacific Northwest National Laboratory Richland Washington USA; ^5^ Soil Biology Group Wageningen University & Research Wageningen the Netherlands

**Keywords:** eco‐evolution, mathematical models, model development, optimality theory, soil carbon cycling, soil microorganisms, soil organic matter

## Abstract

Soil microorganisms mediate carbon and nutrient fluxes in soils, and—as all organisms—are subject to eco‐evolutionary dynamics. Adaptation of soil microbial functionality to environmental conditions across space and time has consequences for biogeochemical fluxes that are often not explicitly considered in models describing soil organic matter (SOM) dynamics. Eco‐evolutionary optimization (EEO) tries to anticipate the outcome of eco‐evolutionary dynamics, and can inform on how microbial functional traits might adapt to environmental conditions based on the maximisation of different proxies of microbial fitness. While different approaches employ different fitness proxies, they all aim to increase realism and generality by grounding SOM models in eco‐evolutionary theory and introducing constraints on model parametrization. Despite this potential, challenges for widely applying EEO approaches to advance SOM models persist and open questions remain, primarily concerning implicit assumptions, convergence of predictions, and empirical validation of the different EEO approaches. In this Synthesis, we review EEO approaches that have been applied to SOM models and provide an instructive primer to EEO approaches. We then propose a general categorization, aiming to make their underlying assumptions explicit and give an outlook for future research directions.

## Graphical abstract

Optimization approaches based on fundamental eco‐evolutionary principles (EEO approaches) can increase the realism and generality of microbial‐explicit soil organic matter (SOM) models—yet, these approaches are not widely used in such models. We review EEO approaches, give a primer to their implementation in SOM models and propose a classification that highlights their implicit assumptions. Challenges persist and we propose a roadmap for further developing EEO approaches in SOM models, highlighting the need for comparative studies.

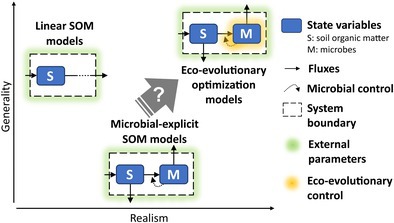

## 1 Introduction

**FIGURE 1:**
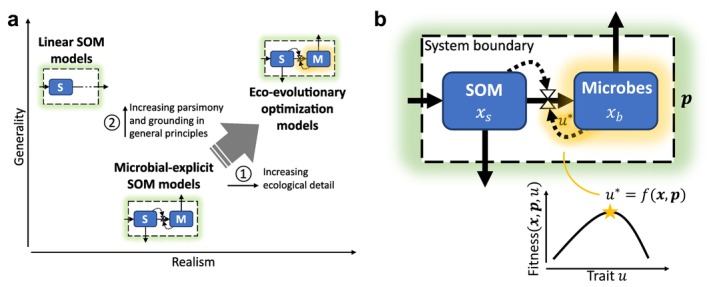
The potential role of eco‐evolutionary optimization approaches in advancing soil organic matter (SOM) modelling. (a) locates ‘traditional’ linear, microbial‐explicit and eco‐evolutionary optimization SOM models in a Realism‐Generality space. Generality incorporates model reliability and robustness (Levins 1966; Prentice et al. 2015). Small illustrations abstract from panel (b) (S, SOM; M, Microbes). (b) box‐flow illustration of a minimalist microbial‐explicit SOM model subject to eco‐evolutionary optimization. Solid arrows represent fluxes, valve symbols indicate non‐linear fluxes and dashed arrows connecting to valves indicate the non‐linear controls. Blue boxes represent state variables (x), green shading represents parameters (p) that are defined outside the system bounds (indicated by the dashed box) and orange shading represents a functional trait (u) that is under eco‐evolutionary selection and attains an optimal value $u^{*}$ (Section 2.3). Colours are consistent with Figure 3.

Microorganisms drive soil nutrient and carbon cycling. Significant efforts have been invested in representing them explicitly in soil organic matter (SOM) models (Chandel et al. 2023; Manzoni and Porporato 2009; Sulman et al. 2018), also at the Earth System scale (Hararuk et al. 2015; Treseder et al. 2012; Wieder, Grandy, et al. 2015; Wieder, Allison, et al. 2015). Unlike traditional linear SOM models, microbial‐explicit models represent how microbial abundance and characteristics shape biogeochemical fluxes and turnover rates (Figure 1; see Chandel et al. 2023 and Sulman et al. 2018 for comprehensive overviews of relevant microbial processes). Detailing these processes increases model realism and can improve predictions, for example, of global soil carbon stocks (Abramoff et al. 2022; Hararuk et al. 2015; Wieder et al. 2013). However, the increased complexity of these models brings about new challenges. Some issues, such as divergent predictions of SOM fate (Sulman et al. 2018; Wieder et al. 2018) might be overcome with the increasing availability of data and harmonised calibration approaches (Tao et al. 2024). Other fundamental issues such as equifinality and parameter identification problems (Marschmann et al. 2019; Sierra et al. 2015; Wieder, Allison, et al. 2015) as well as oscillations and instability (Georgiou et al. 2017; Schwarz et al. 2024; Wang et al. 2014) instead call for a revisiting of existing modelling approaches (Lennon et al. 2024).

Current microbial‐explicit SOM models describe microbial processes with empirically derived rate constants and modifiers more akin to chemical reactions than biological processes. However, microbial properties are not fixed as microbial community composition changes through time and microbial functions can adapt through genetic mutations within populations (see e.g., Abs et al. 2023; Abs, Chase, et al. 2024; Martiny et al. 2023; Wallenstein and Hall 2012 and references therein), giving rise to eco‐evolutionary dynamics (Abs, Chase, et al. 2024; Loreau et al. 2023; Martiny et al. 2023). Explicitly considering aspects of such eco‐evolutionary controls of biotic model components has been proposed to advance SOM modelling (Abs, Chase, et al. 2024; Buchkowski et al. 2017; Treseder et al. 2012), but also to help tackle some of the specific outstanding issues in SOM modelling (Georgiou et al. 2017; Schwarz et al. 2024).

Eco‐evolutionary dynamics can be introduced into models through microbial functional traits that are encoded as model parameters (Van Den Berg et al. 2022). Functional traits are phenotypic characteristics of microorganisms that influence their fitness (Martiny et al. 2015; Violle et al. 2007) and, in the context of SOM modelling, affect ecosystem processes (effect traits; Martiny et al. 2015; Violle et al. 2007; see Table 1 for some examples). A change in functional traits due to microbial adaptation can thus have direct consequences for biogeochemical fluxes (Abs et al. 2023; Schimel and Schaeffer 2012). In SOM models, the values of specific parameters that represent functional traits (e.g., maximal uptake rate constants or half‐saturation constants) can for example be used to distinguish microbial functional groups (e.g., *r*‐ and *K*‐strategists; Wieder et al. 2014). Individual‐based models (IBM) take this approach one step further by explicitly simulating microbial diversity and eco‐evolutionary processes such as competition, selection or dispersal at a fine spatial scale (e.g., Abs, Coulette, et al. 2024; Allison 2012, 2014; Kaiser et al. 2014). However, IBMs are computationally intensive and upscaling to ecosystem‐ or global‐scale processes remains an outstanding challenge (Abs, Coulette, et al. 2024; Wan and Crowther 2022).

Eco‐evolutionary optimization (EEO) approaches provide a toolbox for simplifying model representation of eco‐evolutionary dynamics and allow for their integration into large‐scale models, as recognised in vegetation modelling (Franklin et al. 2020; Harrison et al. 2021). Under the premise that due to natural selection and community assembly (i.e., the combined effect of ecology and evolution) a community may be dominated by the fittest organisms under given environmental conditions, EEO tries to anticipate the outcome of eco‐evolution rather than modelling it explicitly (Franklin et al. 2020). This approach thereby largely abstracts from the complexity of real communities where functionally equivalent taxa may coexist and neutral or stochastic processes can drive shifts in community composition. While minimalistic, EEO provides a first order approximation of which functionally relevant trait might dominate under given conditions (Franklin et al. 2020) and describes a shift in microbial community functioning similar to an emerging response function that guides microbial dynamics along an optimal fitness trajectory. Different from classical response functions (or heuristic arguments) that prescribe a priori how a trait or rate varies with an environmental variable, in EEO approaches response functions emerge from an EEO principle. While this might seem like a mere technical distinction, it constitutes a fundamental shift in viewing microbial functionality: instead of considering microbial functional properties as an additional external control of SOM dynamics (akin to other model parameters p, green shading in Figure 1), EEO conceptualises them as an emergent part of the system (orange shading in Figure 1).

In vegetation models, EEO has allowed for important advances (Harrison et al. 2021), yet EEO approaches remain less developed for integrating microbial adaptation into SOM models. Including EEO considerations into microbial‐explicit SOM models could address some of the persisting challenges when incorporating these models into larger scale models (Figure 1a). Primarily, acknowledging in models that microbes acclimate and adapt to varying environmental conditions in time and space increases realism when considering ecosystem responses to environmental changes (Abs, Chase, et al. 2024; Treseder et al. 2012; arrow 1 in Figure 1a, orange highlights in Figure 1b). EEO can also help constrain model parameters (Franklin et al. 2020). Although new methods utilising, for example genomic data to estimate specific functional traits are now available (Karaoz and Brodie 2022; Li et al. 2025; Marschmann et al. 2024), some model parameters remain challenging to estimate at relevant spatial and organisational scales (Lennon et al. 2024; Wan and Crowther 2022). By assuming that the environment and competitive pressure select microbial communities that are optimally adapted to their environment, EEO approaches offer a top‐down constraint to variations in model parameters related to microbial functional traits—which can decrease the degrees of freedom and increase model parsimony (Chakrawal et al. 2024; Harrison et al. 2021; arrow 2 in Figure 1a). Reducing the number of free parameters can lower precision in matching observed data, yet is required to make models more reliable and robust (Prentice et al. 2015) and to increase model generality and realism (Levins 1966).

EEO approaches are employed in various related fields such as vegetation modelling (Franklin et al. 2020; Harrison et al. 2021), evolutionary biology (Brännström et al. 2013; McGill and Brown 2007; Parker and Maynard Smith 1990; Van Den Berg et al. 2008), biotechnology (Patnaik 2000; Tsiantis and Banga 2020) and in flux balance analysis in genome‐scale models (Dukovski et al. 2021; Edwards et al. 2002; Orth et al. 2010). In microbial‐explicit SOM modelling, various EEO approaches have been applied, yet efforts in this field remain scattered with comparative studies of EEO approaches being the exception and a general theoretical framework still lacking. Here, we give an overview of the different EEO approaches applied in microbial‐explicit SOM models. We first provide a primer on how to set up an EEO approach for such models (Section 2) and then propose a general categorization of common EEO approaches based on their key assumptions (Section 3). We review relevant studies together with an illustrative toy model and show how results differ based on where in our categorization an EEO is located (Section 4). Based on this review, we probe how much of the theoretical potential of EEO approaches for advancing SOM modelling has been explored, discuss persisting challenges for implementing EEO approaches into SOM models and highlight where our framework can aid and where further research is required.

## 2 A Primer on Eco‐Evolutionary Optimization for SOM Modelling

Three components are necessary to incorporate an EEO approach in microbial‐explicit SOM models: (1) adaptable functional traits that independently have relevance for microbial fitness as well as for biogeochemical fluxes, (2) trade‐offs that constrain the adaptation of the functional traits and (3) a mathematical framework that quantifies fitness depending on the trade‐offs in a functional trait and environmental conditions.

### 2.1 Functional Traits to Link Eco‐Evolutionary Dynamics and Matter Fluxes

In the context of SOM modelling, relevant functional traits are phenotypic characteristics that affect fitness and simultaneously have an effect on biogeochemical fluxes (Martiny et al. 2015; Violle et al. 2007) (Table 1), thus linking eco‐evolutionary dynamics to biogeochemical cycles (Wallenstein and Hall 2012). In turn, fitness is the net outcome of costs and benefits of a microbial strategy, and is thus linked to matter and energy flows through the microbial biomass (Abs et al. 2023). Although evolutionary processes act at the level of individuals or populations, most SOM models represent the microbial community as a single, aggregated pool. In this context, functional traits are best interpreted as community‐aggregated traits (CATs; Fierer et al. 2014) and adaptation at the community level as shifts in CATs (Wallenstein and Hall 2012).

**TABLE 1:** Examples of microbial functional traits used in soil organic matter models employing eco‐evolutionary optimization (EEO) approaches. The listed constraints are not always explicitly accounted for in the cited publications.

Functional trait	Constraint	Example EEO models
Uptake rate	Mass balance; Thermodynamics	Manzoni et al. 2023
Substrate use efficiency	Thermodynamics (Gibbs free energy production)	Allison 2014; Manzoni et al. 2017
Extracellular enzyme investment	Mass balance (resource investment into enzymes vs. other functions is limited by total acquired resources)	Abs et al. 2020, 2025; Bonner et al. 2022; Calabrese et al. 2022; Chakrawal et al. 2024; Vetter et al. 1998
Allocation between enzyme classes (targeting different substrates/nutrients)	Mass balance (resource investment into specific enzymes is limited by total acquired resources)	Averill 2014; Wutzler et al. 2024

### 2.2 Trade‐Offs to Constrain Eco‐Evolution

No organism can maximise all functions simultaneously, but faces trade‐offs (Wallenstein and Hall 2012). While trade‐offs can be motivated by heuristic arguments or empirical observations, in microbial‐explicit models they are usually linked to mass and energy conservation principles and also apply to CATs. Classically, trade‐offs between fast and inefficient versus slow and efficient growth have been considered, for example, by considering *r*‐ versus *K*‐strategists, respectively copiotrophs versus oligotrophs (e.g., Pagel et al. 2020; Wieder et al. 2014), or between metabolic pathways (e.g., fermentation vs. respiration; Pfeiffer and Bonhoeffer 2002). More complex trade‐off frameworks have also been proposed, for example, the Yield‐Acquisition‐Stress tolerance (YAS) framework of Malik, Martiny, et al. (2020).

**FIGURE 2:**
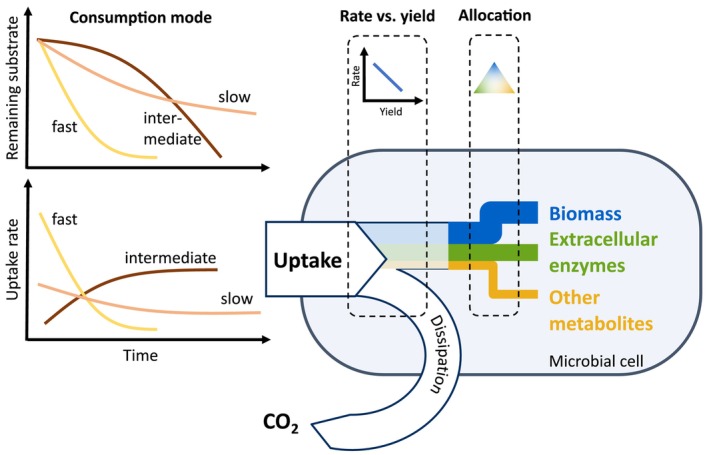
Examples of how trade‐offs in functional traits are embedded in microbial‐explicit soil organic matter models. The illustration of the rate versus yield trade‐off is based on Calabrese et al. (2021) and that of the allocation trade‐off is based on Malik, Martiny, et al. (2020) and Piton et al. (2023).

Mass balance directly constrains the allocation between metabolic products: for example, the same unit of substrate that is used to produce extracellular enzymes cannot be used to also produce biomass or any other compound. Microbial consumption strategies are also constrained by mass balance: a rapid uptake rate might be advantageous if competing for a finite public resource; however, the entailing resource depletion will limit microbial growth later on. Therefore, a trade‐off emerges between uptake rate and the duration for which fast growth on a finite resource can be sustained. These trade‐offs (Figure 2) are implicit in the mass balance equations for microbial resources that can be included in the optimization framework. Trade‐offs can also be derived from thermodynamic constraints. For example, a rate‐yield trade‐off can be motivated thermodynamically as the speed of a chemical reaction is proportional to the free energy it dissipates, which allows for less energy being stored in reaction products (Figure 2; Pfeiffer and Bonhoeffer 2002; Westerhoff et al. 1983).

Trade‐offs between functional traits imply that there is a continuum of possible strategies (i.e., combination of connected trait values) with associated costs and benefits and a resulting fitness continuum that depends on the environment. EEO approaches identify optimal trait values that correspond to the fitness maximum for given conditions and assume that lumped community dynamics are effectively dominated by organisms that express these optimal traits: as any organism is successively outcompeted by yet a fitter organism, natural selection will shift CATs towards a fitness maximum.

### 2.3 A Mathematical Framework to Quantify Fitness

We can express trade‐offs in terms of adaptable fitness costs $C\left(E,u\right)$ and benefits $B\left(E,u\right)$ associated with a specific value of the focal trait u in a given environment E (all mathematical symbols are described in Table 2). These trade‐offs in traits emerge because any fitness benefit comes at a cost. To compute the fitness associated with a functional trait value u in the environment E, an objective function $O\left(E,u\right)$ is used to weight the fitness costs $C\left(E,u\right)$ and benefits $B\left(E,u\right)$. For example, by producing extracellular enzymes at a rate u, microbes have to pay a metabolic cost for production of those enzymes $C\left(E,u\right)$, but also gain some benefit $B\left(E,u\right)$ in terms of an increased amount of available substrate. The form of the functions $B\left(E,u\right)$ and $C\left(E,u\right)$ depends on the considered functional trait, its trade‐offs and its feedback with the environment (see examples in Box 1). $O\left(E,u\right)$ in turn depends on the assumptions made about what is eco‐evolutionarily optimised. Parker and Maynard Smith (1990) gave a comprehensive summary of the basic construction of such a function. We build on this framework by separating the effects of the chosen fitness measure and optimization timescale, which gives rise to our classification of EEO approaches (Section 3).

**TABLE 2:** Definition of symbols used throughout the manuscript.

Symbol	Definition
B	Benefit to microorganisms dependent on adaptable traits
C	Cost to microorganisms dependent on adaptable traits
$f\left(\cdot \right)$	Generic functional relation
K	Sum of all non‐adaptable costs and benefits ($K=K^{\prime }-D$, where D are non‐adaptable mortality terms)
O	Objective function to be maximised or minimised
p	Vector of model parameters, not adaptable (e.g., environmental and constant physiological parameters)
t	Time
T	Terminal time
$u,u_{r},u_{m}$	Adaptable trait value (of the resident $\left(r\right)$ and mutant $\left(m\right)$, respectively)
x	Vector of state variables
X	Vector of steady state solutions of state variables
$x_{b}$	Biomass of microbial species
$x_{s}$	Resource/Soil organic matter
μ	Net specific growth rate
$\kappa_{u},\kappa_{p},\kappa_{x}$	Characteristic rate (inverse of the characteristic time) of microbial trait adaptation, changes in environmental parameters and ecological feedback, respectively
Superscript *	Optimal solution
Subscript 0	Initial condition
*Symbols used in examples and the toy model*	
$c_{0}$	Parameter quantifying competitive advantage of enzyme producers
d	Microbial mortality rate coefficient [d^−1^]
$d_{e}$	Enzyme decay rate coefficient [d^−1^]
I	SOC input rate [mg C g^−1^ d^−1^]
$k_{u}$	Half‐saturation constant of the decomposition/growth rate [mg C g^−1^]
$\ell_{s}$	Abiotic loss rate coefficient of SOC [d^−1^]
$m_{e},m_{e}^{r},m_{e}^{m}$	Biomass‐specific microbial enzyme production/release rate (of the resident $\left(r\right)$ and mutant $\left(m\right)$, respectively) [d^−1^]
q	Parameter group in the example of Calabrese et al. (2022)
$r_{b},r_{e}$	Respiration rate coefficients of maintenance ($r_{b}$) and enzyme production ($r_{e}$) [d^−1^]
$v_{u}$	Maximum rate coefficient of the growth/decomposition rate [d^−1^]
y	Microbial yield, the fraction of decomposition rate that is not respired [1]
α	Fraction of reactive SOC [1]
β	Fraction of SOC that is microbial biomass C [1]
$\varepsilon_{e}$	Cost factor of enzyme production [1]
ϕ	Fraction of microbial assimilation rate used for enzyme production in Abs et al. (2025) [1]
ρ	Enzymatic/microbial decomposition rate of SOC [mg C g^−1^ d^−1^]

In microbial‐explicit SOM models, the environment E is defined by parameters $p=\left(p_{1},p_{2},\ldots ,p_{n}\right)$ (all parameters that cannot be adapted by microbes) and the state variables $x=\left(x_{1},x_{2},\ldots ,x_{n}\right)$ (e.g., the microbial biomass and SOM content, which we always consider to be the minimal set of state variables for these models) (Figure 1b); p and x might vary in time. The objective function can be written as

$$\begin{array}{c} \overset{\begin{array}{c} \text{Objectivefunction}\\ \text{to}\;\mathrm{be}\;\text{maximized} \end{array}}{\overbrace{O\left(x,p,u\right)}}=f\left(\;\overset{\begin{array}{c} \text{Adaptable}\\ \text{benefits} \end{array}}{\overbrace{B\left(x,p,u\right)}},\underset{\begin{array}{c} \text{Adaptable}\\ \text{costs} \end{array}}{\underbrace{C\left(x,p,u\right)}},\overset{\begin{array}{c} \text{Non‐adaptable}\\ \text{costs}\;\text{and}\;\text{benefits} \end{array}}{\overbrace{K\left(x,p\right)}}\right) \end{array}$$

Next to the identification of a relevant functional trait and its associated trade‐off, the definition of the objective function O is a key choice in using EEO.

## 3 Fitness Measures and Timescales to Classify EEO Approaches

To allow comparisons across studies, we classify EEO approaches based on how they quantify fitness—that is, on the assumptions that are embedded in the objective function $O\left(x,p,u\right)$. Similarly, in evolutionary biology, frequency‐dependent and ‐independent approaches are contrasted (Parker and Maynard Smith 1990). We distinguish two main dimensions of $O\left(x,p,u\right)$ that vary among EEO approaches and can lead to systematically different predictions: (1) the fitness measure, that is, the metric used to approximate fitness and (2) the timescale at which the optimal response is determined. This categorization helps to understand the implicit ecological assumptions associated with the different EEO approaches and how they affect the optimization results.

### 3.1 Common Fitness Measures

#### 3.1.1 Net Specific Growth Rate

A first approximation of microbial fitness might be the number of offspring per individuum. In SOM models, this is often approximated by increases in microbial biomass (e.g., measured as mg of microbial biomass C per g of soil). Based on the assumption that microbes reproduce asexually and have a constant cell mass, the number of offspring per individuum corresponds to the net biomass growth per unit biomass over time—that is, the net specific growth rate μ. We refer to approaches that maximise μ as growth maximisation approaches.

Following the definition of the objective function $O\left(x,p,u\right)$, μ can be expressed in terms of some costs $C\left(x,p,u\right)$ and benefits $B\left(x,p,u\right)$ for microbial fitness associated with a functional trait u. In Box 1 we apply this framework to the example of Calabrese et al. (2022) who analysed the optimal production of extracellular enzymes to release a growth substrate. Alternative assumptions can lead to various flavours of this approach. For instance, Averill (2014) assumed a fixed microbial enzyme investment but that microbes can flexibly allocate this investment between enzymes that target different substrates and so obtain different benefits. In that framework, only adaptable benefits B and fixed costs K exist, but no adaptable costs (C=0). The choice of an optimal trait value then depends on the opportunity cost (i.e., the lost profit) emerging from choosing one strategy over the other—investment into enzyme 1 reduces the amount of enzyme 2 being produced.

BOX 1 **Common fitness measures in eco‐evolutionary optimization approaches.**

We consider microbial mass balance dynamics of the form

$$\frac{1}{x_{b}}\frac{dx_{b}}{dt}=\underset{\text{Adaptable}}{\underbrace{B\left(x,p,u\right)-C\left(x,p,u\right)}}+\underset{\text{Non‐adaptable}}{\underbrace{K\left(x,p\right)}}=\overset{\text{Net}\;\text{specific}\;\text{growth}\;\text{rate}\;\mu }{\overbrace{B\left(x,p,u\right)-C\left(x,p,u\right)+K\prime \left(x,p\right)}}-\overset{\text{Mortality}}{\overbrace{D\left(x,p\right)}}$$

distinguishing between adaptable terms (that are functions of the focal trait u) and non‐adaptable terms (that are not a function of u), B representing benefits and C costs. $K\left(x,p\right)=K^{\prime }\left(x,p\right)-D\left(x,p\right)$ sums non‐adaptable benefits and costs (benefits enter with positive and costs with negative sign). Following convention in microbial‐explicit modelling (Manzoni et al. 2012) B,C and $K^{\prime }$ consist of uptake, respiration and exudation terms while non‐adaptable microbial mortality D is considered separately. Often D=const is assumed, but D might also be a function of x, for instance, if density‐dependent mortality is considered.

The **net specific growth rate** is given by

$$\mu =B\left(x,p,u\right)-C\left(x,p,u\right)+K^{\prime }\left(x,p\right)$$

The marginal fitness change $\frac{\partial \mu }{\partial u}$ quantifies whether there is a fitness gain or loss from increasing the value of u. The optimal strategy $u^{*}$ is found from

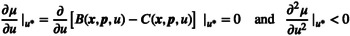

where 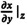 is the partial derivative of x with respect to y evaluated at z (where $\frac{\partial }{\partial u}K^{\prime }\left(x,p\right)=0$); meaning that $u^{*}$ is a maximum of μ.

Following this approach, Calabrese et al. (2022) investigated the optimal microbial production rate of extracellular enzymes $u≔m_{e}$ in a system similar to that in Figure 1b where microbes require extracellular enzymes to degrade SOC into an available substrate they can use for growth. They define the net specific growth rate as

$$\mu =\underset{\text{Adaptable benefit}}{\underbrace{\overset{\text{Growth rate}}{\overbrace{yv_{u}\frac{m_{e}x_{s}^{2}}{m_{e}x_{s}^{2}+q}}}}}-\underset{\text{Adaptable cost}}{\underbrace{\overset{\text{Enzyme production}}{\overbrace{m_{e}\left(1+r_{e}\right)}}}}-\underset{\text{Non‐adaptable}}{\underbrace{\overset{\text{Maintenance}}{\overbrace{r_{b}}}}}$$

where $p=\left(q,r_{b},r_{e},v_{u},y\right)$ are fixed parameters and the SOC content $x_{s}$ is a state variable. Setting 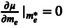 Calabrese et al. (2022) found the optimal strategy to be a direct function of the SOC content: $m_{e}^{*}=\sqrt{\frac{q}{1+r_{e}}}x_{s}^{-1}-\frac{q}{yv_{u}}x_{s}^{-2}$ which they approximated as $m_{e}^{*}\approx \sqrt{\frac{q}{1+r_{e}}}x_{s}^{-1}$.

**Invasion fitness** expands the fitness concept by considering the appearance of a rare mutant strain $x_{m}$ that varies slightly in its trait value $u_{m}$ compared to a resident population (which has biomass $x_{r}$ and the trait value $u_{r}$) (Dieckmann and Law 1996; Geritz et al. 1998; Metz et al. 1995). The mutant mass balance is given by

$$\frac{1}{x_{m}}\frac{dx_{m}}{dt}=B\left(x,p,u_{m}\right)-C\left(x,p,u_{m}\right)+K^{\prime }\left(x,p\right)-D\left(x,p\right)$$

where $x=\left(x_{r},\ldots \right)$ is the vector of state variables excluding the mutant biomass. As the mutant is considered to be rare ($x_{m}\ll x_{r}$), it does not affect the system dynamics in its initial growth phase. The mutant is assumed to appear only after the resident population has attained its ecological equilibrium, meaning the system is at steady state with the resident population ($\frac{dx}{\mathrm{dt}}=0$, with the solutions 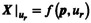). The invasion fitness $s\left(u_{m},u_{r}\right)$ then assesses if the rare mutant has a positive growth rate in this environment that is set by the resident and whether it can consequently invade

$$s\left(u_{m},u_{r}\right)={\left(\frac{1}{x_{m}}\frac{dx_{m}}{dt}\right)}_{x=X_{u_{r}}}=B\left(p,u_{m},u_{r}\right)-C\left(p,u_{m},u_{r}\right)+K^{\prime }\left(p,u_{r}\right)-D\left(p,u_{r}\right)\;\left\{\begin{array}{c} >0\\ =0\\ <0 \end{array}\;\begin{array}{c} \text{mutant}\;\mathrm{can}\;\text{invade}\\ \text{for}\;u_{m}=u_{r}\\ \text{mutant cannot invade} \end{array}\right.$$

A successfully invading mutant then becomes the new resident and a new ecological equilibrium is reached before the next mutant appears (compare illustration in Table 3). Notice that by substituting the steady state solutions 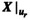, $s\left(u_{m},u_{r}\right)$ is independent of the state variables (see further details in Section 3.2 and Box 2). Fitness extrema $u^{*}$ are found by setting 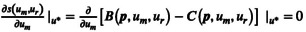, (where $\frac{\partial K^{\prime }\left(p,u_{r}\right)}{\partial u_{m}}=\frac{\partial D\left(p,u_{r}\right)}{\partial u_{m}}=0$) evaluating the partial derivative at $u^{*}=u_{m}=u_{r}$ and solving for $u^{*}$. An $u^{*}$ that additionally satisfies 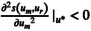 and 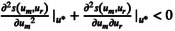 at $u^{*}=u_{m}=u_{r}$ is termed a continuously stable strategy (CSS) and represents an endpoint of evolution (see Brännström et al. 2013; McGill and Brown 2007 for comprehensive reviews). The first condition thereby ensures that $u^{*}$ is a maximum (evolutionarily stable) and the second that evolution converges to this point (Eshel 1983; Metz et al. 1995).

Abs et al. (2025) followed this approach to investigate how, in a similar system as in Calabrese et al. (2022), microbial investment into extracellular enzyme production might evolve if cheaters can emerge in a population due to mutation. To this aim, they defined the invasion fitness as a function of the relative investment into extracellular enzyme production (u≔ϕ), the steady state concentration of available substrate defined by the resident, and a function that quantifies the competitive advantage (disadvantage) of a mutant that produces more (less) enzymes than the resident (to capture the effect of spatial heterogeneities in an implicit way). An optimal $\phi^{*}$ that is resistant to invasion by a nearby mutant and a CSS was found as $\phi^{*}=1-\frac{d}{yv_{u}}-\frac{1}{c_{0}}$ where $c_{0}$ is a new parameter quantifying the competitive advantage of enzyme producers.

Parameter symbols of both examples were adapted from the originals to be comparable throughout this work and are described in Table 2. Further details on both examples are reported in the Supporting Information Section 2.

When applying growth maximisation to these models with a single microbial pool, it is implicitly assumed that competition is negligible—or alternatively, that all microbes cooperate by sticking to the same strategy. This assumption breaks down under conditions where competition affects microbial fitness and deviant strategies, such as opportunistic ‘cheating’ can emerge.

#### 3.1.2 Invasion Fitness

The invasion fitness framework explicitly considers cases where deviant strategies can emerge. Invasion fitness is the fitness measure in adaptive dynamics, which is derived from evolutionary and game theory principles (Brännström et al. 2013; Dieckmann and Law 1996; Geritz et al. 1998; McGill and Brown 2007; Metz et al. 1995). It is different from the net specific growth rate, as it does not measure an absolute fitness quantity, but the fitness advantage of a rare mutant (characterised by having a slightly different trait value) relative to a resident population at equilibrium (Box 1). Adaptive dynamics assumes that if a mutant has a positive invasion fitness while it is still rare, it will replace the resident and become the new resident itself before a new mutant appears. This framework can predict evolutionarily singular strategies with many alternative outcomes and interpretations, such as evolutionarily stable strategies or branching points (Geritz et al. 1998; Metz et al. 1995). In the context of SOM modelling, a single convergent and evolutionarily stable equilibrium—known as a continuously stable strategy (CSS, Eshel 1983; Metz et al. 1995)—may be the most practical outcome (Abs et al. 2025), as system dynamics can otherwise become more complex making it difficult to predict a single steady‐state carbon stock.

Two limitations of this approach are particularly relevant for SOM models: the assumption of continuous small mutation steps, which may not hold when modelling eco‐evolutionary processes rather than strict evolution, where trait changes can be larger; and the assumption of rare mutations, which imposes a strict separation of timescales—ecological dynamics (e.g., resource and biomass changes) are faster than trait change (Dieckmann and Law 1996; Geritz et al. 1998) (compare Box 2). These assumptions may break down in rapidly changing environments or in systems with fast dispersal.

#### 3.1.3 Heuristic Approaches

Some studies are based on heuristic optimality arguments. They are motivated by microbial fitness considerations (and often economic reasoning)—but rather than optimizing a defined fitness measure, they assume a priori what strategy should be optimal. For instance, Sinsabaugh and Moorhead (1994) assumed that the ‘optimal’ abundance of nutrient acquiring enzymes is inversely proportional to nutrient availability; while Weverka et al. (2023) and Wutzler et al. (2017) assumed that allocation between enzymes should be proportional to their return on investment. In this Synthesis we focus on formal optimization approaches, yet we note that under some conditions, heuristic approaches can yield similar results (Wutzler et al. 2024).

### 3.2 Timescale of Optimization

**FIGURE 3:**
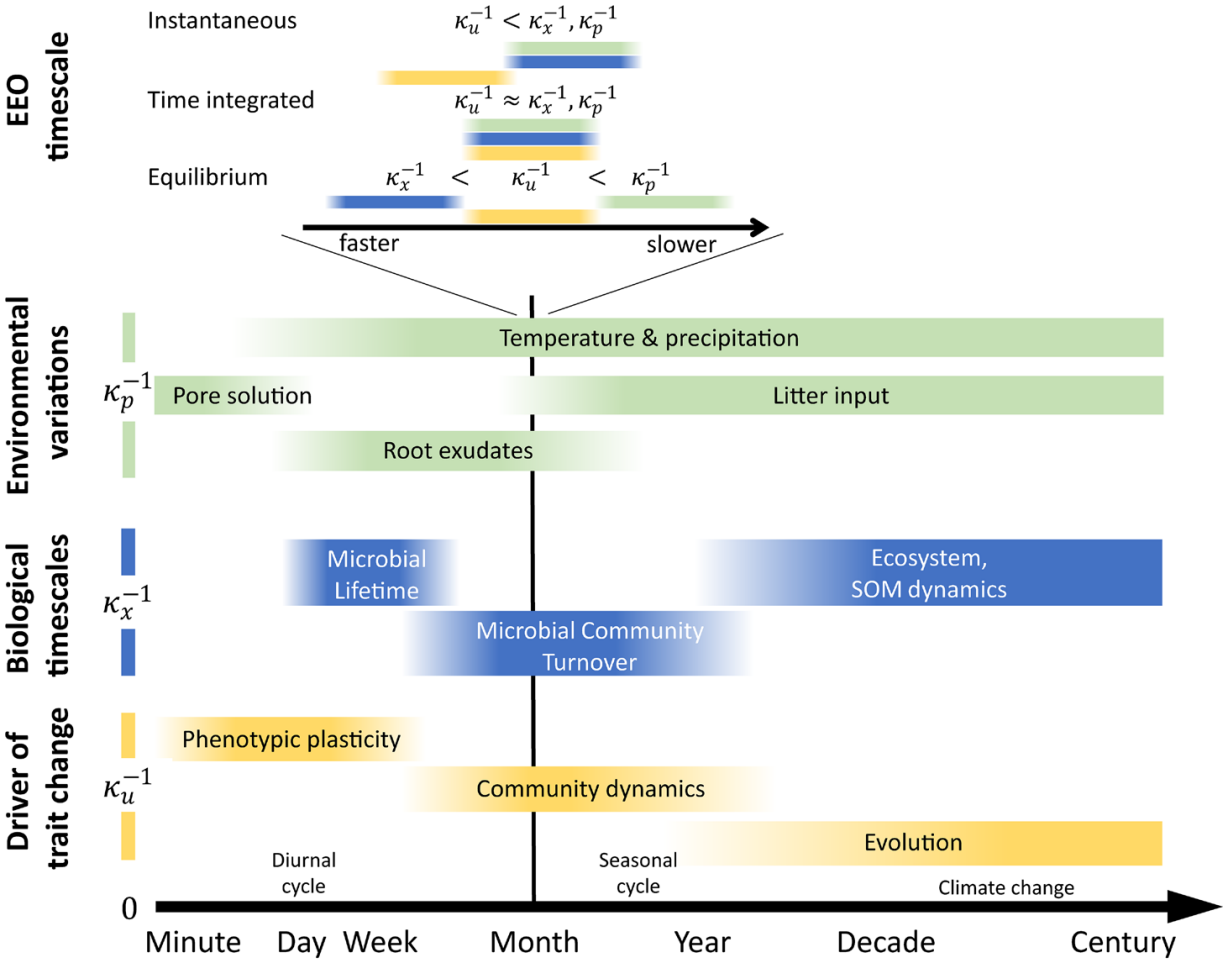
Schematic comparing timescale assumpitons of eco‐evolutionary optimization (EEO) approaches to environmental and biological timescales (Abramoff et al. 2018; Abs, Chase, et al. 2024; Abs, Coulette, et al. 2024; Martiny et al. 2023). EEO timescales are not absolute, but relative to the assumptions about the characteristic rates of different processes ($\kappa_{p},\kappa_{x},\kappa_{u}$; their inverse indicating characteristic timescales). These assumptions are illustrated by the coloured bars at the top of the figure for an arbitrary characteristic timescale (black vertical line).

In addition to defining an appropriate fitness measure, the relevant timescales for optimization needs to be identified. This choice carries an assumption about the relative timescales of the ecological feedback (the dynamics of the state variables; $\kappa_{x}=\frac{1}{x}\frac{dx}{dt}$), environmental change (the dynamics of the external parameters; $\kappa_{p}=\frac{1}{p}\frac{dp}{dt}$), and changes in the focal microbial trait ($\kappa_{u}=\frac{1}{u}\frac{du}{dt}$) (Wallenstein and Hall 2012). We might for example consider the characteristic rate of change in a microbial trait to be faster than that of ecological feedbacks and environmental change ($\kappa_{u}>\kappa_{x},\kappa_{p}$)—for example, if we assume that community‐level adaptation in a trait is dominated by phenotypic plasticity and we are interested in long‐term changes in SOM dynamics with variations in litter input (Figure 3). Such assumptions are carried in the optimization timescale and have direct implications for the outcome of the optimization problem. In the previous example, the fast microbial adaptation of the trait u would be in a dynamic equilibrium with respect to the slower changes in state variables or environmental parameters. In other words, on timescales on which we observe changes in x or p (e.g., SOM content or litter fall), the trait u would always already be adapted to these new conditions. This assumption about the optimization timescale also decides the mathematical form of the optimal $u^{*}$ and whether $u^{*}$ is a function of model parameters only, or also depends on the state variables.

#### 3.2.1 Instantaneous Optimization

Instantaneous optimization assumes that at every instant in time a functional trait adapts and becomes optimal, exclusively according to the current environment. Therefore, an instantaneous optimization strategy does neither anticipate nor account for its long‐term impact on the environment. By following this strategy, organisms might thus create less favourable conditions for themselves or their offspring in the future. The conditions experienced by the organism (the environment E) are defined by the external parameters (p) and for example, the substrate availability (defined by a state variable in x). Thus, in this approach, state variables can (but do not have to) appear in the definition of the optimal trait (Box 2). Accordingly, the optimal trait can vary in synchrony with the state variables (and/or changes in other parameters).

BOX 2 **Timescales of eco‐evolutionary optimization approaches.**

***Instantaneous:** **Ecological feedback slower than changes in microbial traits*** ($\kappa_{x},\kappa_{p}<\kappa_{u}$)

In instantaneous optimization problems, the objective function is given by

$$O=f\left(x,p,u\right)$$

and the sought optimization outcome can be written as

$$u^{*}=f\left(x,p\right)$$

which means that changes in the optimal trait value are determined by the values of x and p. For instance, as p changes, so does $u^{*}$. Therefore, this approach implies that microbial traits react faster to environmental changes than the ecological feedback: at the timescale on which p (or x) changes, $u^{*}$ has already adapted to this new condition.

The example of Calabrese et al. (2022) described in Box 1 is an example of instantaneous optimization.

***Time integrated:** **Changes in microbial traits as fast as ecological feedback*** ($\kappa_{u}\approx \kappa_{x},\kappa_{p}$)

For time integrated optimization, the objective function is given as the integral of the objective function until a terminal time T

$$O=\int_{0}^{T}f\left(x\left(t\right),p\left(t\right),u\left(t\right)\right)\;dt$$

This approach yields the optimal trajectory $u^{*}\left(t\right)$ for $t=\left[0,T\right]$ and the initial condition $x_{0}=x\left(t=0\right)$ as

$$u^{*}\left(t\right)=f\left(x_{0},p,t,T\right)$$

The solution for $u^{*}$ can in some cases be converted from the time to the state variable domain and $u^{*}$ can be re‐expressed as

$$u^{*}\left(t\right)=f\left(x\left(t\right),p\left(t\right)\right)$$

which is in the same form as in instantaneous optimization, but bound to a given temporal trajectory $t=\left[0,T\right]$ where T might be a function of $x_{0}$. This approach implicitly assumes that microbes have been selected so as to anticipate what happens over the time interval $\left[0,T\right]$.

For instance, Manzoni et al. (2023) assessed the optimal rate of microbial decomposition $u\left(t\right)≔\rho \left(t\right)$ of a given amount of carbon substrate (see details in Supporting Information Section 2). With an optimal control approach, they assessed how $\rho \left(t\right)$ needs to change during the period it takes to decompose all the substrate to maximise the cumulative microbial growth over that period. All parameters p were kept constant and the temporal dynamics emerged from the predicted dynamics of the adaptive trait $\rho \left(t\right)$ and the state variables $x\left(t\right)$. Even though time integrated optimization implies time‐varying traits (i.e., a non‐autonomous system of equations), in some cases these traits can be linked analytically to the state variables (thus recovering an autonomous system). In the previous example, Manzoni et al. (2023) found that, when neglecting maintenance respiration, the optimal decomposition kinetics scaled as the square root of the substrate content, and the optimal growth kinetics resembled a Hill function with exponent ½.

***Equilibrium:** **Changes in microbial traits slower than ecological feedback*** ($\kappa_{p}<\kappa_{u}<\kappa_{x}$)

In equilibrium optimization, state variables (x) are substituted with their respective steady states ($X=f\left(p,u\right)$), yielding an objective function as

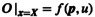

and the sought optimal traits can be written as

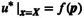

In this approach, the steady state X is fully determined by the environmental conditions p and the trait u; and the optimal trait $u^{*}$ is determined by the environmental conditions p. This might be interpreted as microbes shaping their environment as to maximise their long‐term fitness. Dependent on the model structure and type of EEO, the steady state assumption might face analytical limitations. For a homogeneous population/community (no mutants/invaders) at steady state the net specific growth rate μ is equivalent to the mortality rate 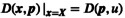 (since 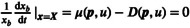; Box 1). If microbial mortality is assumed to be linear so that D=const, it follows that μ=D=const is independent of u and no optima can be calculated. However, with adaptive dynamics, in the presence of a mutant whose growth is not balanced by mortality, trait optima can be found. This is the case of the example described in Box 1 from Abs et al. (2025).

Figure 3 graphically illustrates the assumptions on relative timescale relations in the different EEO approaches.

#### 3.2.2 Time Integrated

Criticising instantaneous optimization approaches, Vallino (2010) argued that in contrast to abiotic chemical reactions, biological systems integrate strategies over time and so have evolved to ‘predict the future’ (Vallino 2010, p. 1419). This notion is captured by time integrated fitness measures (Box 2) that evaluate fitness over a given time period, for example, the life‐time of an individual, several generations, or some characteristic time of the environment (Figure 3). In this approach, the effect of a strategy on the environment and its reciprocal effect on microbial fitness over a finite time period is accounted for.

This class of problems are solved using optimal control theory (Kirk 1970; Lenhart and Workman 2007). If we consider that u can change with time, we can find an optimal temporal trajectory $u^{*}\left(t\right)$ from t=0 to some terminal time T. Similar optimal control approaches are used in genome‐scale metabolic models to optimise cellular strategies over time (Tsiantis and Banga 2020). In SOM modelling, optimal control approaches have been used to assess the temporal progression of litter degradation (Box 2; Chakrawal et al. 2024; Manzoni et al. 2023).

If environmental conditions change unpredictably or over a range of timescales ($p\left(t\right)$; in non‐autonomous systems), it could be more informative to assume that microbes adapt to the stochastic regime of environmental variations. The state variables would then be represented as probability density functions instead of deterministic values. Such a stochastic modelling approach has been proposed in vegetation models to describe plant responses to variable soil moisture (Cowan 1986; Lu et al. 2016), and Van Den Berg et al. (2008) assessed optimal microbial resource allocation considering stochastic arrival of competitors, but to our knowledge this approach has not been applied to optimise microbial traits in SOM models.

#### 3.2.3 Equilibrium

Similar to time integrated optimization, equilibrium optimization considers how a microbial trait influences the environment (the state variables) and how this in turn affects microbial fitness; though not along a specific temporal trajectory, but once the environment has equilibrated with such a change. This introduces a notion of co‐evolution between microbes and their environment (Dieckmann and Law 1996): eco‐evolutionary dynamics lead to the selection of those microbes that are the fittest in the environment they (co‐)create. The equilibrium approach thereby assumes that changes in traits occur slowly in comparison to ecological feedbacks ($\kappa_{u}<\kappa_{x}$) so that, for example, microbial biomass can reach its equilibrium before a further trait change occurs. This time‐scale separation is formalised in adaptive dynamics by contrasting slow genetic mutation with fast population dynamics (Dieckmann and Law 1996; Geritz et al. 1998).

In equilibrium optimization, all state variables are set to their steady state values $X=f\left(p,u\right)$, determined from solving $\frac{dx}{dt}=0$. The objective function then only depends on environmental parameters p, and the adaptable trait u (Box 2). For instance, Vetter et al. (1998) determined substrate fluxes transported to a microbe thanks to steady state concentration gradients to assess the optimal enzyme release rate. Usually, the assumption of steady state is not absolute, but it is required that the variables reach a dynamic equilibrium quickly relative to changes in u or p. The steady state assumption can introduce unintuitive equalities, as for example, microbial growth would always need to be balanced by mortality or other biomass losses. The steady state assumption also is a basis for flux balance analysis, an optimization approach used in genome‐scale modelling in which intracellular metabolic fluxes are assumed to rapidly equilibrate with the external conditions (Edwards et al. 2002; Orth et al. 2010).

## 4 EEO Applications to SOM Models, Their Challenges and Ways Forward

We first present a brief overview of relevant studies that have implemented EEO approaches into SOM models, highlighting general themes and directions of previous research efforts. We then illustrate how our classification framework can help to understand differences in model predictions and how it might help to identify underlying processes. Lastly, we discuss persisting challenges and offer a roadmap for addressing them.

### 4.1 A Brief Overview

EEO approaches have been used to assess how eco‐evolutionary dynamics affect diverse microbial traits in various settings (Table 1, Supporting Information Table S1). Microbial (extracellular) enzyme production meets both criteria of a suitable functional trait—sensitivity to environmental change and importance to biogeochemical cycles (Allison et al. 2010; Schimel and Weintraub 2003)—and appears as a common target in many EEO studies. EEO studies have addressed, for example, how much enzymes microbes should produce as a function of available SOC (e.g., Calabrese et al. 2022) or enzyme and hydrolysate diffusion (Vetter et al. 1998), what types of enzymes to produce under varying carbon and nutrient availabilities (Averill 2014; Wutzler et al. 2024), or how competition can affect enzyme production (Abs et al. 2025; Bonner et al. 2022). While time integrated optimization and adaptive dynamics can become analytically cumbersome, instantaneous growth rate maximisation might be more straightforward to implement and is often used in microbial‐explicit SOM modelling studies (Table 3, Supporting Information Table S1). Usually, a single optimization objective is defined for the whole microbial community—with the exception of Smith and Wan (2019) who explicitly distinguished saprotrophic and ectomycorrhizal fungi in their model, resulting in different formulations for optimal growth in the two fungal groups.

**TABLE 3:** Eco‐evolutionary optimization approaches mapped to the proposed classification reveals implicit assumptions. Guide to illustrations: Lines represent temporal trajectories of an optimised fitness measure. Inserts indicate the fitness measure as a function of the trait u and locate the optimal trait value $u^{*}$. $\kappa_{x}^{-1}$ indicates the characteristic timescale of ecological feedback (cf. Box 2). In the adaptive dynamics panel $u_{r}$ and $u_{m,i}\;\left(i=1,2,\ldots ,n\right)$ indicates the resident, respectively the mutant traits, illustrating repeated successful (+) and unsuccessful (−) invasion events (signs representing the sign of the mutant invasion fitness). Successful invasion leads to fixation of the trait $u_{m}\to u_{r}$ which by definition has zero invasion fitness compared to itself.

Fitness measure	Optimization timescale		
	Instantaneous $\;\kappa_{x},\kappa_{p}<\kappa_{u}$	Time integrated $\kappa_{x},\kappa_{p}\approx \kappa_{u}$	Equilibrium $\kappa_{p}<\kappa_{u}<\kappa_{x}$
**Net specific growth rate**	Instantaneous Maximization 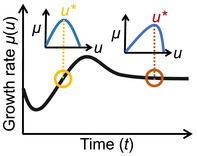 Fitness only dependent on current environment and focal trait. Examples: Allison 2014; Averill 2014; Calabrese et al. 2022	Optimal Control 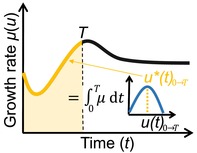 Fitness dependent on (temporally variable) environment, focal trait, and how it shapes environment over the specified time period. Examples*:* Chakrawal et al. 2024; Manzoni et al. 2023	Equilibrium Maximization 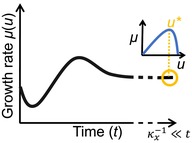 Fitness dependent on environment, focal trait, and how it shapes the environment in the long term. Example*:* Vetter et al. 1998
**Invasion fitness**			Adaptive dynamics 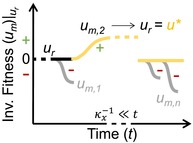 Fitness dependent on resident population, resident and mutant traits, and how they shape the environment. Examples*:* Abs et al. 2020, 2025;

Many studies are of conceptual nature and aim to deepen process understanding or build hypotheses. For example, Bonner et al. (2022) derived the hypothesis that, under certain conditions, enzymatic degradation of available SOM by fungi might be inhibited by the presence of ‘cheating’ bacteria. Others contrast predictions against experimental data of litter mass loss (Chakrawal et al. 2024; Manzoni et al. 2023), element ratios of substrates and organisms (Manzoni et al. 2017), CUE (Calabrese et al. 2022; Manzoni et al. 2017), and per‐biomass enzyme activities (Calabrese et al. 2022); but also microbial DNA and RNA contents (Franklin et al. 2011). EEO models were successful in predicting patterns in the data that are not captured by standard models, such as a negative correlation between per‐biomass potential enzyme activity and SOC content (Calabrese et al. 2022), and performed equally well as standard models in data fitting after model calibration, despite having fewer free parameters (Chakrawal et al. 2024; Manzoni et al. 2023). However, challenges remain for systematically advancing SOM models using EEO approaches.

### 4.2 Convergence of Different Eco‐Evolutionary Optimization Approaches

**FIGURE 4:**
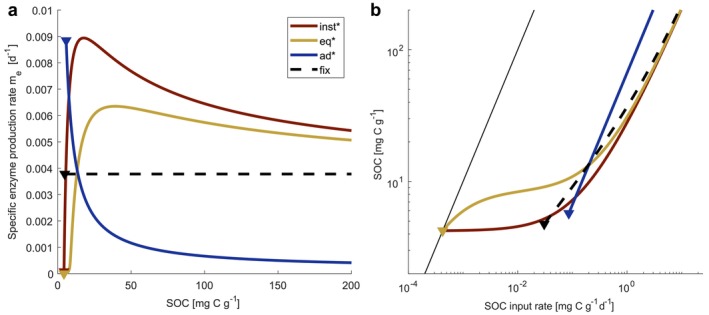
Emergent relations from different eco‐evolutionary optimization (EEO) approaches in the toy model (Box 3). (a) Optimal per‐biomass extracellular enzyme production rate as a function of SOC content obtained from different EEO approaches ($m_{e}$, with: inst*, instantaneous maximisation; eq*, equilibrium maximisation; ad*, adaptive dynamics; see Box 3) compared to a ‘traditional’ fixed (fix) parametrization. Only in the instantaneous maximisation $m_{e}^{*}$ is a direct function of SOC; in the other EEO approaches variations in $m_{e}^{*}$ and SOC are due to variations in the input rate leading to an emergent $m_{e}^{*}$‐SOC relationship. (b) emerging relationship between SOC content and SOC input rate with the different $m_{e}^{*}$. The thin solid black line refers to a system without microbes ($m_{e}=0$). Inverted triangles mark substrate levels below which approaches yield unphysical results.

Even if assessing similar processes, results and insights might differ as a consequence of using different EEO approaches. Understanding these differences motivates our classification of EEO approaches along two axes: fitness measure (Section 3.1) and optimization timescale (Section 3.2) (Table 3). We use the studies of Calabrese et al. (2022) and Abs et al. (2025) as examples of optimal enzyme production (Box 1). In both models, microbes produce enzymes optimally to degrade SOC into an available growth substrate. We also introduce a minimal toy model (Box 3, Figure 4), based on the same system, to test different EEO approaches on exactly the same model structure.

BOX 3 **Toy model illustrating EEO approaches.**

Here we apply different EEO approaches (Table 3) to the same minimal toy model. The toy model's structure corresponds to the illustration in Figure 1b representing microbial turnover of SOC but for simplicity neglects some processes relevant for SOC turnover.

SOC dynamics are controlled by external inputs with flux I [mg C g^−1^ d^−1^], enzymatic degradation ρ [mg C g^−1^ d^−1^], and abiotic losses (erosion, leaching, …) with rate $\ell_{s}$ [d^−1^]

(1) $$\frac{dx_{s}}{dt}=I-\rho -\ell_{S}x_{s}$$

where $x_{s}$ [mg C g^−1^] is the SOC content. Microbes have biomass $x_{b}$ [mg C g^−1^] and consume the flux of degradation products ρ with an efficiency y [1], invest into enzyme production, and decay according to the rate coefficient d [d^−1^]

(2) $$\frac{dx_{b}}{dt}=\overset{\text{Growth rate}}{\overbrace{\mathrm{y\rho}}}-\overset{\text{Enzyme production}}{\overbrace{\varepsilon_{e}m_{e}x_{b}}}-\overset{\text{Mortality}}{\overbrace{dx_{b}}}=\mu x_{b}-dx_{b}$$

where $m_{e}$ [d^−1^] is the specific enzyme release rate, $\varepsilon_{e}>1$ [1] the associated respiration costs and μ [d^−1^] is the net specific growth rate. ρ is described by equilibrium chemistry approximation kinetics (Tang and Riley 2013) as

(3) $$\rho =v_{u}\frac{\alpha x_{s}\;x_{e}}{\alpha k_{u}+\alpha x_{s}+x_{e}}$$

where $x_{e}$ [mg C g^−1^] is the extracellular enzyme concentration, $v_{u}$ [d^−1^] is the maximum decomposition rate, $k_{u}$ [mg C g^−1^] the half‐saturation constant and α [1] is the fraction of accessible reactive sites of SOC. Enzymes are released by microbes at rate $m_{e}x_{b}$ and decay at rate $d_{e}x_{e}$, where $d_{e}$ [d^−1^] is the rate constant of enzyme decay. For simplicity and comparability with the example in Calabrese et al. (2022) (Box 1), we assume $x_{e}$ and $x_{b}$ to be at quasi‐steady state and that microbial biomass is a fraction β [1] of SOC: $x_{e}≔X_{e}=\frac{m_{e}}{d_{e}}x_{b}$ and $x_{b}≔X_{b}=\beta x_{s}$. By imposing $\frac{dx_{b}}{dt}=0$, microbial mortality needs to balance growth. Thus, the mortality coefficient becomes a function of other state variables and parameters, $d=d\left(x,p,u\right)$ (compare Equation 2). The model is defined by the parameter vector $p≔\left(\alpha ,\beta ,\varepsilon_{e},d_{e},I,k_{u},\ell_{S},v_{u},y\right)$, the focal trait $u≔m_{e}$, and $x_{s}$ as the only remaining state variable.

Substituting $X_{e}$ and $X_{b}$ into Equation 2 and 3 we obtain μ as

(4) $$\mu =\underset{\text{Adaptable Benefit}}{\underbrace{\overset{\text{Growth rate}}{\overbrace{yv_{u}\frac{\alpha x_{s}\;\frac{m_{e}}{d_{e}}}{\alpha k_{u}+\alpha x_{s}+\frac{m_{e}}{d_{e}}\beta x_{s}}}}}}-\underset{\text{Adaptable Cost}}{\underbrace{\overset{\text{Enzyme prodution}}{\overbrace{\varepsilon_{e}m_{e}}}}}$$

In **instantaneous maximisation** of the net specific growth rate, SOC ($x_{s}$) is considered as constant compared to the timescale needed to adapt $m_{e}$. Thus, we find the optimal microbial enzyme production rate $m_{e}^{\text{inst}*}$ by solving $\frac{\partial \mu }{\partial m_{e}}=0$ for $m_{e}$ and checking that 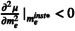. As expected from our framework (Box 2), $m_{e}^{\text{inst}*}$ is a function of parameters p and the state variable $x_{s}$.

For **equilibrium maximisation** of the net specific growth rate, we first find the steady‐state SOC concentration $X_{s}$ from setting $\frac{dx_{s}}{dt}=0$ (Equation 1). By substituting $x_{s}≔X_{s}$ in Equation 4 we obtain 
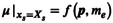
. We then find $m_{e}^{\mathrm{eq}*}$ from solving 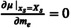 and checking that 
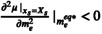
. $m_{e}^{\mathrm{eq}*}$ differs from $m_{e}^{\text{inst}*}$ as it is not a function of $x_{s}$ but p only, including parameters describing SOC fate (I and $\ell_{s}$). As $X_{s}$ is a function of $m_{e}$ the optimal strategy $m_{e}^{\mathrm{eq}*}$ does account for its own effect on substrate availability.

For **adaptive dynamics** we assess the invasion fitness $s\left(m_{e}^{r},m_{e}^{m}\right)$ of a rare mutant $x_{m}$ with trait $m_{e}^{m}$ that appears in an environment that is at equilibrium with a resident $x_{b}$ that has trait $m_{e}^{r}$ (i.e., the environment is fully defined by that resident and given by the steady state solutions 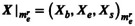 and $x_{m}\ll X_{b}$). The resident's growth dynamics at equilibrium is given by Equations (2), (3), (4) as
(5)

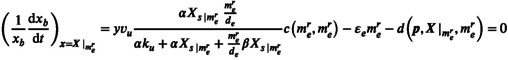

which we expanded by the term $c\left(m_{e}^{r},m_{e}^{r}\right)$ [1], accounting for preferential access to depolymerization products ρ to those that produce more enzymes (Abs et al. 2025). As all microbes in the resident population produce the same number of enzymes, no one has a benefit and $c\left(m_{e}^{r},m_{e}^{r}\right)=1$. Thus, this extension does not affect any of the previous results. However, a mutant could produce more enzymes than the resident population to gain greater access to depolymerization products ($c\left(m_{e}^{r},m_{e}^{m}\right)>1$ for $m_{e}^{m}>m_{e}^{r}$), while cheaters have reduced access ($c\left(m_{e}^{r},m_{e}^{m}\right)<1$ for $m_{e}^{m}<m_{e}^{r}$). In analogy to the resident dynamic (Equation 5), the mutant's invasion fitness $s\left(m_{e}^{r},m_{e}^{m}\right)$ is found as
(6)

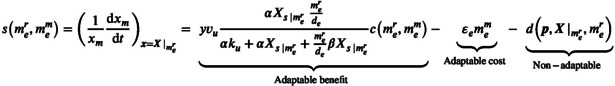

We find a continuously stable strategy (Box 1) $m_{e}^{\mathrm{ad}*}$ by solving 
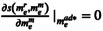
 at $m_{e}^{m}=m_{e}^{r}=m_{e}^{\mathrm{ad}*}$ and checking that 
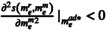
 and 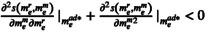 at this $m_{e}^{m}=m_{e}^{r}=m_{e}^{\mathrm{ad}*}$ (Box 1). Like $m_{e}^{\mathrm{eq}*}$ also $m_{e}^{\mathrm{ad}*}$ is a function of only parameters p, though with one additional unknown parameter 
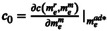
 that quantifies how much producing more enzymes increases the access to depolymerization products relative to the resident.

Figure 4 illustrates how optimal trait values vary across SOC levels. Analytical solution and further details are given in Supporting Information Section 3. The MATLAB code of the toy model is available at https://doi.org/10.5281/zenodo.15148570.

Results of Calabrese et al. (2022) and Abs et al. (2025) differ along the two axis of our classification framework (Table 3). First, they differ in their *fitness measure*: the adaptive dynamics approach in Abs et al. (2025) emphasises microbial competition—consequently parameters that determine the competitive advantage become determinant for the optimal strategy (Box 1). Instead, maximising the net specific growth rate in Calabrese et al. (2022) implicitly assumes that microbial competition is negligible and thus parameters related to microbial interaction do not affect the optimal strategy. Second, they differ in their *optimization timescale*: in the instantaneous maximisation approach of Calabrese et al. (2022) microbes adapt to the environment they are currently experiencing (defined by some parameters p and the current substrate availability $x_{s}$) without considering the effect they have on the environment. Consistent with our classification, this yields the optimal strategy as a function of the SOC content (a state variable). Instead, in the adaptive dynamics approach used by Abs et al. (2025), which optimises at the equilibrium timescale, state variables are replaced by their corresponding steady state expressions. The effect that microbes have on their environment is thus accounted for in the optimal strategy and optimal enzyme allocation is a function of some microbial parameters and the competitive advantage, but is independent of any state variable. The results of our toy model analysis mirror these systematic differences between EEO approaches (Box 3; Supporting Information Section 3).

What do these differences imply for model predictions? In our toy model, instantaneous and equilibrium growth maximisation approaches differ only slightly, with the equilibrium approach predicting a consistently lower extracellular enzyme investment. This leads to greater accumulation of SOC at a given SOC input rate (Figure 4b) and indicates that in the long run, if competition is negligible, it is beneficial to reduce consumption especially at low input rates (while both approaches converge at very high input rates; Supporting Information Section 3). Similarly, Manzoni et al. (2023) observed in their time integrated optimization approach that optimal microbial litter degradation diminishes if competition with other processes causing resource loss is reduced. All approaches we tested (including the adaptive dynamics approach) predict a decrease in the biomass‐specific enzyme production with increasing SOC (at SOC > 50 mg C g‐soil^−1^) (Figure 4a). In turn, both growth maximisation approaches predict a sharp decrease of the specific enzyme production towards very low SOC contents—which agrees with the exact analytical solution of Calabrese et al. (2022) (Box 1)—but is absent in the adaptive dynamics approach. This leads to very different predictions of SOC accumulation at low input rates (Figure 4b) and can be explained by the different fitness measures. As SOC content declines, more enzymes are required to sustain depolymerization while simultaneously microbial uptake declines. Growth maximisation approaches aim to maximise the difference between increasing costs for enzyme production and the decreasing uptake rate (i.e., the net specific growth rate). Thus, enzyme production is decreased to sustain growth at low SOC (Figure 4). In contrast, invasion fitness emphasises the relative difference between mutant and resident growth rate rather than growth rate per se. Even if a strategy yields an overall lower specific growth rate once established, it is selected if it provides a relative fitness advantage to a rare mutant in an environment dominated by a resident with a slightly different strategy. In the adaptive dynamics approached applied in our toy model, primary access to degradation products is always granted to those microbes that produce relatively more enzymes. At low SOC content, where degradation products are scarce, this becomes a relevant fitness advantage, resulting in higher specific enzyme production at low SOC (Figure 4a).

Taken together, these results illustrate that while common patterns can emerge, EEO results are sensitive to their underlying, often implicit, assumptions. Caution is thus required when drawing conclusions from individual approaches and models, while comparative studies can identify patterns emerging consistently across approaches (Levins 1966; Templeton and Lawlor 1981). Divergent EEO predictions suggest testable hypotheses—for example, does microbial enzyme investment at low substrate concentrations stall or increase further and can this be explained by competition effects?

Currently, studies that compare EEO approaches in SOM models are rare. Notably, Wutzler et al. (2024) compared a heuristic and a formal instantaneous growth maximisation approach to extracellular enzyme production. They found the former to be a special case of the more formal optimization approach and results consequently converged under some conditions. However, a rigorous comparison between other EEO approaches in SOM models and between model structures is still missing. General mathematical treatments of when adaptive dynamics and growth maximisation approaches converge exist (Gyllenberg et al. 2011; Metz et al. 2008), and have been applied to epidemiological models (Dieckmann and Metz 2006; Lion and Metz 2018), but to our knowledge not in microbial‐explicit SOM modelling. Our toy model and the examples from the literature illustrate that the analytical solutions of EEO approaches vary predictably along the two axes of our classification—fitness measure and optimization timescale. While this framework remains qualitative, it can aid interpretation and guide future systematic comparison studies.

### 4.3 Validation of EEO Predictions

Insights derived from EEO approaches need to be contrasted with relevant empirical evidence. However, our goal is not to prove assumptions about microbial adaptation or fitness‐dependent selection (see Abs et al. 2023; Abs, Chase, et al. 2024; Martiny et al. 2023; Wallenstein and Hall 2012 for compilations of empirical examples), but to assess whether the outcomes of EEO‐based models match observed patterns. In some cases, a direct comparison of data and an optimized trait is possible. For instance, Manzoni et al. (2017) showed that an adaptable microbial CUE that maximises the instantaneous growth rate at the community level could predict the observed sensitivities of CUE to substrate C:N and C:P, under different levels of N and P fertilisation and in both aquatic and terrestrial systems. Widely available data such as time series of litter mass loss can instead be too unspecific to allow selecting a modelling approach in calibration tests (Abs, Chase, et al. 2024; Chakrawal et al. 2024; Manzoni et al. 2023). While more specific empirical measures often do not directly align with modelled quantities, normalised trends in related proxies (e.g., the model predicted microbial enzyme investment and the measured potential enzyme activity per unit microbial biomass; Calabrese et al. 2022) can be compared. For example, time series of enzyme activities can be used in time‐integrated optimization (Chakrawal et al. 2024), whereas activities obtained from spatial sampling across pedoclimatic gradients are more suitable for instantaneous optimization (Calabrese et al. 2022). Abs et al. (2025) used various measurements (e.g., metagenomics, enzyme activities) from the literature to qualitatively validate predictions of their adaptive dynamics approach. Data sets from long‐term field experiments with manipulations of environmental conditions or inputs to the soil could be useful to evaluate predictions from equilibrium EEO approaches.

Additionally, metagenomic data could be converted into ‘potential’ traits at a high level of functional detail, and thus be valuable for the validation of EEO approaches and for the testing of derived hypotheses (Abs, Chase, et al. 2024; He et al. 2024). For instance, aggregated potential traits of soil bacterial communities derived from shotgun metagenomes vary to some degree predictably with soil environmental factors (Piton et al. 2023), though limitations in the database required for annotation complicate interpretation (Osburn et al. 2024; Piton et al. 2024). Kinetic parameters such as half‐saturation constants and specific respiration rates can be predicted from metagenome‐assembled genomes using the computational pipeline *microTrait* (Karaoz and Brodie 2022) coupled with a dynamic energy budget model (DEBmicroTrait; Marschmann et al. 2024) (Li et al. 2025). A bias towards known genes and functions remains (Maxeiner et al. 2023) and technical difficulties and associated costs when determining soil microbial genome assembly (Anthony et al. 2024) are bottlenecks to the integration of metagenomic data in microbial‐explicit models. Harmonisation efforts, leveraging existing metagenomic databases, might be able to fill these gaps in the future (Anthony et al. 2024). Once available, these data may be best employed in validating EEO approaches to ground models in an empirically substantiated process understanding rather than as parameter values (Franklin et al. 2020) in standard microbial‐explicit models.

### 4.4 Technical Challenges for Implementation in Ecosystem Models

While EEO approaches have the potential to parsimoniously integrate eco‐evolutionary controls on SOM turnover into ecosystem models, their implementation might face technical challenges. For instance, Zhang et al. (2018) implemented a heuristic approximation for flexible microbial CUE under varying nitrogen or carbon limiting conditions derived from an EEO approach (instantaneous growth maximisation; Manzoni et al. 2017) into an extension of the CENTURY model (Parton et al. 1987). While the adapting trait improved model performance, its implementation required the introduction of new parameters that needed calibration (Zhang et al. 2018). This example points to two persisting challenges for implementing EEO approaches in ecosystem models, which are further developed below: (1) EEO approaches may not be (easily) transferable between models; and (2) EEO approaches do not necessarily increase model parsimony (Figure 1).

EEO solutions may be found analytically, numerically or graphically (e.g., Metz et al. 1995; Vetter et al. 1998). Analytical solutions are appealing as they can yield direct insights into parameter relations and can help increase model parsimony. Yet, they are usually not transferable to other model formulations and are limited to relatively simple systems and optimization problems. Wutzler et al. (2024) recently argued for using numerical derivative‐based approximations of formal optimality criteria. For their model, such an approach closely matched results of a formal instantaneous growth rate maximisation approach without requiring cumbersome analytical solutions. Specifically, their ‘derivative approach’ assumes the focal trait to evolve along the marginal fitness gradient, that is, increasing (decreasing) an investment as long as there is a marginal gain (loss) until an optimal strategy is reached.

Opposite to what we would expect, in practice EEO approaches do not necessarily increase model parsimony. This is not only the case if approaches are transferred to a different model (as in Zhang et al. 2018) but also if EEO approaches invoke processes not previously included in a model. For example, the adaptive dynamics approach of Abs et al. (2025) as well as our toy model implementation of an adaptive dynamics approach (Box 3) required the introduction of a new unconstrained parameter that quantifies the competitive advantage of enzyme producers. While this can point to missing processes in the original model, it does not reduce the number of free parameters.

Other examples show that EEO approaches can indeed be used to constrain not just a single microbial parameter, but an entire biological function. For instance, Manzoni et al. (2023) used optimal control theory to constrain the microbial substrate uptake rate, whose exact mathematical form and parameters are uncertain. They found an optimal uptake rate that resembles the classical Monod function and could fit data equally well, though without any of the assumptions implicit in the Monod kinetics (Box 2, Supporting Information Section 2). Similarly, Chakrawal et al. (2024) showed that despite having fewer parameters than a traditional model, with an optimal control approach their EEO model performed equally well against data and provided additional insights about the onset of ligninolytic activity that were not accessible from a traditional model.

There is no clear indication of which EEO approach works best to constrain free parameters in a given model formulation. Yet, existing SOM model formulations can be systematically analysed to test if some of their parameters can be optimised via an EEO approach. The requirements are outlined in Section 2: (1) a functional trait that can be interpreted as affecting microbial fitness and (2) a trade‐off that can be expressed in terms of costs and benefits for an organism's fitness and thus constrains trait variation. Our Synthesis provides an overview of how to construct a relevant EEO approach from these components.

Lastly, we note that adapting microbial traits could in theory avoid undesired instabilities or reduce oscillations in microbial‐explicit SOM models (Schwarz et al. 2024). However, we are not aware of any study that has tested how EEO approaches affect stability properties in SOM models. Our toy model indicates that compared to a ‘standard’ modelling approach with fixed microbial parameters, both instantaneous and equilibrium growth maximisation approaches could extend the range of conditions (the substrate input rate) for which the model yields a positive microbial growth rate and is thus applicable (Figure 4b). A systematic assessment of the mathematical properties of EEO‐based models could facilitate their further integration into ecosystem models.

Given the conceptual and technical challenges outlined in Section 4, we propose a roadmap for further developing EEO approaches for microbial‐explicit SOM models:

1. Systematically compare outcomes of different EEO approaches and test for consistent patterns (Section 4.2)
2. Validate predicted trait values with trait estimates and emerging relations among variables or fluxes using empirical data matching the modelled quantities (Section 4.3)

To further leverage the potential of EEO approaches for ecosystem modelling, researchers should

3 Tackle persisting technical limitations by exploring transferable and less cumbersome approaches (Section 4.4)
4 Integrate models and EEO approaches when (Section 4.4):
  - existing models already contain the components required for the implementation of an EEO approach
  - a specific EEO approach increases model parsimony and/or stability

### 4.5 Conceptual Frontiers in Developing EEO Approaches for Microbial‐Explicit SOM Models

To allow analytical tractability, most optimization approaches target a single trait or trait‐related trade‐off (but see e.g., Franklin et al. (2011) and Wutzler et al. (2024), e.g., with more complex trade‐offs). However, traits can co‐vary according to life‐history strategies and trade‐offs between several traits might be connected (Malik, Martiny, et al. 2020; Malik, Swenson, et al. 2020; Piton et al. 2023). Expanding the space of adaptable traits can quickly lead to mathematically intractable problems. As the number of independent traits increases, numerical solutions become necessary.

Also, the diversity of ‘soil microorganisms’ and its simplistic representation in SOM models poses a persistent challenge for the reliability of EEO approaches. Most current models group bacteria, fungi and archaea into a single pool to parsimoniously parametrize models (Crowther et al. 2019). By applying an optimization approach to this pool of ‘soil microbes’, it is assumed that all of these organisms adhere to the same optimization criteria. Yet, bacteria follow a fundamentally different life form than for example, plant symbiotic fungi and might thus have different traits under selection. Fine‐scale taxonomic distinctions are probably not informative for SOM modelling, but microbes might be grouped into coarse‐grained functional groups (Lennon et al. 2024). While some models, such as MIMICS (Wieder et al. 2014), use different parameter values to distinguish among groups, from an EEO perspective we might instead distinguish them by selecting different adaptable traits depending on their life form (as e.g., in Smith and Wan 2019).

An alternative perspective to tackle these challenges is provided by the maximum entropy production (MEP) approach. Derived from the principle that a system self‐organises to maximise the production of entropy, a maximisation approach can be applied to the sum of all dissipating reactions within a system—an idea already proposed by Lotka (1922). This approach can extend to entire food webs (e.g., Vallino 2010) or ecosystems (Dewar 2010). MEP approaches can accommodate several control variables, for instance traits of multiple interacting organisms (e.g., Vallino 2010). However, microbial communities are not just heterogeneous with respect to the identity of their members, but also in their microscale spatial distribution and hence their capability to interact with each other (Raynaud and Nunan 2014). While the full breadth of the complexity of soil microbial ecology cannot be captured in any optimization approach, approaches such as (spatially resolved) individual‐based models (IBM) are valuable tools to investigate scenarios that are outside of the assumptions of EEO approaches (Abs, Chase, et al. 2024; Abs, Coulette, et al. 2024; Allison 2014). Insights from these studies can then give guidelines on how to further develop EEO approaches.

In a broader perspective, a range of optimization approaches are applied at various levels of organisation: from genome‐scale for flux balance analysis, to populations or communities with negligible competition for growth rate maximisation or competition with mutants for adaptive dynamics, to trophic networks, communities or whole ecosystems for MEP. Future research might aim at integrating these approaches to advance SOM modelling across spatial and ecological organisation scales—though unifying these scales in SOM modelling remains an outstanding challenge in its own (Wan and Crowther 2022). In all such efforts it is important to remember that the real world might not be as strict in optimising a specific target as mathematical approaches suggest, and evolution instead embraces some level of imperfection (Bejan 2023).

## 5 Conclusion

Several studies have implemented eco‐evolutionary optimization (EEO) approaches in soil organic matter (SOM) models, leading to new conceptual insights and predictions for SOM fate that differed from those of conventional models. Together these studies showcase the potential of EEO approaches to advance SOM modelling by incorporating ecological knowledge. Specifically, EEO allows microbial traits to adapt to a range of environmental conditions based on eco‐evolutionary principles, without prescribing how microbial functional traits should change a priori. In doing so, these approaches can foster deeper process understanding and make models more parsimonious, while maintaining predictive performance comparable to traditional models.

However, these potentials could not always be realised, pointing to some challenges. Poor transferability of EEO approaches between model formulations can be restrictive. Here heuristic approximation approaches (Wutzler et al. 2024) might help to make EEO approaches more accessible to the general soil modelling community. More fundamentally, even with similar model formulations, different EEO approaches can yield systematically different results for optimal traits that maximise fitness under given conditions.

Our framework can help to understand systematic differences in EEO approaches by making assumptions on the timescale and type of optimization explicit. Systematic comparative studies in combination with validation against empirical data are needed to identify and confirm relations that emerge as consistent between EEO approaches, and to further develop an understanding of which EEO approach is most suitable under given conditions. Other questions concerning EEO applicability, for example, whether the mathematical properties of these models make incorporation into Earth system models more feasible, still await investigation. Addressing these challenges can inform the new generation of ecology‐aware SOM models.

## Author Contributions

**Erik Schwarz:** conceptualization, investigation, writing – original draft, writing – review and editing, visualisation. **Elsa Abs:** conceptualization, writing – review and editing. **Arjun Chakrawal:** writing – review and editing. **Luciana Chavez Rodriguez:** writing – review and editing. **Pierre Quévreux:** writing – review and editing. **Stefano Manzoni:** conceptualization, investigation, writing – review and editing, visualisation, funding acquisition, supervision.

## Funding

This project has received funding from the European Research Council (ERC) under the European Union (EU)'s Horizon 2020 Research and Innovation Programme (project SMILE, grant agreement 101001608) and under the EU's Horizon Europe Programme (project GAMEchange, grant agreement 10116464), and by Schmidt Sciences LLC (project CALIPSO). Views and opinions expressed are however those of the author(s) only and do not necessarily reflect those of the EU or the ERC Executive Agency. Neither the EU nor the granting authority can be held responsible for them.

## Supporting information


**Table S1:** Summary of eco‐evolutionary optimization approaches applied (or applicable) to soils, including information on: mechanisms each work focuses on, selected adaptable trait, constraints or trade‐offs, objective of the optimization, time scale of optimization and approach (AD, adaptive dynamics; Heuristic, implementation of a heuristic measure; Max, growth rate maximisation; MEP, maximum entropy production; OC, optimal control).
**Table S2:** Parameter values used in toy model simulations. If not indicated differently, parameter values are taken from ranges and ‘baseline’ parameter values in Schwarz et al. (2024), who complied parameter values from Cotrufo and Lavallee (2022), Hararuk et al. (2015) and Tao et al. (2023).

## Data Availability

The Matlab code to run the toy model is available from the Zenodo Digital Repository: https://doi.org/10.5281/zenodo.15148570. No data were used in this article.
