# Supplementary material for: Eco‐Evolutionary Optimality in Soil Organic Matter Models
Source: Ecol Lett. 2025 Dec 19;28(12):e70278. doi: 10.1111/ele.70278 (PMC12717351; doi:10.1111/ele.70278)
Supplement: Supplementary file 1 — Table S1: Summary of eco‐evolutionary optimization approaches applied (or applicable) to soils, including information on: mechanisms each work focuses on, selected adaptable trait, constraints or trade‐offs, objective of the optimization, time scale of optimization and approach (AD, adaptive dynamics; Heuristic, implementation of a heuristic measure; Max, growth rate maximisation; MEP, maximum entropy production; OC, optimal control). Table S2: Parameter values used in toy model simulations. If not indicated differently, parameter values are taken from ranges and ‘baseline’ parameter values in Schwarz et al. (2024), who complied parameter values from Cotrufo and Lavallee (2022), Hararuk et al. (2015) and Tao et al. (2023). [file ELE-28-0-s001.docx]

Supporting Information:

**Eco-evolutionary optimality in soil organic matter models**

Erik Schwarz^1,2,^*, Elsa Abs^3^, Arjun Chakrawal^4^, Luciana Chavez Rodriguez^5^, Pierre Quévreux^1,2^, Stefano Manzoni^1,2^

^1^ Department of Physical Geography, Stockholm University, Stockholm, Sweden

^2^ Bolin Centre for Climate Research, Stockholm University, Stockholm, Sweden

^3^ Laboratoire des Sciences du Climat et de l’Environnement, Saint-Aubin, France

^4^ Environmental Molecular Sciences Laboratory (EMSL), Pacific Northwest National Laboratory, Richland, Washington, USA

^5^ Soil Biology Group, Wageningen University & Research, Wageningen, The Netherlands

*Corresponding author: erik.schwarz@natgeo.su.se

# **Summary table of eco-evolutionary optimization approaches**

Table S1 Summary of eco-evolutionary optimization approaches applied (or applicable) to soils, including information on: mechanisms each work focuses on, selected adaptable trait, constraints or trade-offs, objective of the optimization, time scale of optimization, and approach (AD: adaptive dynamics; Heuristic: implementation of a heuristic measure; Max: growth rate maximization; MEP: maximum entropy production; OC: optimal control).

| **Source** | **Mechanism** | **Adaptable trait (control)** | **Constraint or trade-off** | **Objective** | **Time scale** | **Approach** |
| --- | --- | --- | --- | --- | --- | --- |
| Averill (2014) | Allocation to enzymes for C or N acquisition | Allocation to production of different enzymes | Constant total enzyme allocation | Max resource acquisition rate | Instantaneous | Max |
| Calabrese *et al.* (2022) | Extracellular enzyme production for C acquisition | Enzyme release rate | Cost of enzyme production | Max growth rate | Instantaneous | Max |
| Wutzler *et al.* (2024) | Allocation to enzymes for C, N or P acquisition | Allocation to production of different enzymes | Constant total enzyme investment | Max total return | Instantaneous | Max |
| Allison (2014) | Thermal adaptation | Carbon use efficiency | Growth rate-yield tradeoff | Max growth rate | Instantaneous | Max |
| Manzoni *et al.* (2017) | Allocation under varying nutrient availability | Carbon use efficiency | Nutrient availability | Max growth rate | Instantaneous | Max |
| Heaton *et al.* (2016) | Exploitive fungal growth on substrate | Resource consumption rate, growth rate, recycling rate | Available energy budget | Max net growth rate or reproductive effort of fungi | Instantaneous or over a set time period | Max |
| Smith & Wan (2019) | N-source utilization and plant-mycorrhiza C-N trading | Use of labile or recalcitrant N source, plant-directed N transfer | Cost of making N from recalcitrant source available and N-cost of C | Max growth rate | Instantaneous | Max |
| Bonner *et al.* (2022) | Oxidative extracellular enzyme production for C acquisition | Enzyme release rate | Cost of enzyme production; probability that C released cannot be taken up by enzyme producer | Max growth rate | Instantaneous | Max |
| Sinsabaugh & Moorhead (1994) | Allocation to enzymes for C, N, or P acquisition | Allocation to production of different enzymes | Constant total enzyme investment | Max community productivity | Instantaneous | Heuristic |
| Weverka *et al.* (2023) | Intracellular enzyme/ transporter production for substrate utilization | Allocation to production of different enzymes | Constant total enzyme investment | Multiple, e.g., return on investment proportional allocation | Instantaneous | Heuristic |
| Wutzler *et al.* (2017) | Allocation to enzymes for C or N acquisition | Allocation to production of different enzymes | Constant total enzyme investment | Multiple, e.g., return on investment proportional allocation | Instantaneous | Heuristic |
| Parnas & Cohen (1976) | Intra-cellular storage | Synthesis and remobilization of storage | Constant amount of resources | Max cumulative growth rate | Set time period | Max |
| Manzoni *et al.* (2023) | Consumption of finite resource | Resource uptake rate | Decreasing CUE with increasing uptake rate | Max cumulative growth rate | Time to complete decomposition (free terminal time problem) | Max |
| Chakrawal *et al.* (2024) | Oxidation capacity of litter degraders | Rate constant of lignin decomposition | Energetic cost of oxidative enzyme production and maintenance | Max cumulative growth rate | Time to complete decomposition (set a priori) | Max |
| Vallino (2010) | Allocation to metabolic reactions in a foodweb | Allocation to biological structure synthesis and energy coupling | Total biomass constrained by mass balance and thermodynamic constrains | Max entropy production | Set time period | MEP |
| Vetter *et al.* (1998) | Extracellular enzyme production for resource acquisition | Enzyme release rate | Energetic cost of enzyme production; decreasing return on investment due to transport limitation | Max resource acquisition rate | Equilibrium | Max |
| Franklin *et al.* (2011)^a^ | Partitioning  of biomass in uptake, growth, maintenance | Cellular fraction for growth and maintenance | Constant total cell biomass; minimum allocation to structural biomass | Max growth rate | Equilibrium | Max |
| Pfeiffer & Bonhoeffer (2002)^a^ | Rate and yield of ATP production-pathway | ATP yield | Net free energy difference of ATP production-pathway | Payoff for a mutant | Equilibrium | AD |
| Abs *et al.* (2020) | Extracellular enzyme production for resource acquisition | Fraction of  investment in enzyme production | Cost of enzyme production | Invasion fitness of a mutant | Equilibrium | AD |
| Abs *et al.* (2025) | Extracellular enzyme production for resource acquisition | Fraction of  investment in enzyme production; level of competition asymmetry | Cost of enzyme production | Invasion fitness of a mutant | Equilibrium | AD |

^a^ Resource content is considered as a parameter and not a state-variable. The steady-state assumption applies to the rest of the system.

# **Details on literature examples**

Parameter symbols of literature examples were adapted from the originals to be comparable throughout this work.

*Examples presented in Box 1:*

Calabrese *et al.* (2022) assessed the biomass-specific enzyme production rate $m_{e}$ as the focal functional trait ($u:=m_{e}$) in a model of similar structure as Fig 1b where microbes (with biomass $x_{b}$) need to produce extracellular enzymes $x_{e}$ in order to degrade soil organic carbon (SOC) $x_{s}$ into an available substrate (dissolved organic carbon (DOC), $x_{d}$). They described microbial uptake of $x_{d}$ by Monod-type kinetics as

$\frac{1}{x_{b}}\rho=v_{u}\frac{x_{d}}{x_{d}+k_{u}}$

Calabrese *et al.* (2022) further assumed that $x_{d}=\gamma x_{s}\cdot x_{e}$ where $\gamma$ is a proportionality coefficient and that extracellular enzymes are at quasi-steady state so that $x_{e}:=X_{e}=\frac{m_{e}}{d_{e}}x_{b}$, and that $x_{b}$ is in quasi-equilibrium and always equal to a fixed fraction $\beta$ of SOC: $x_{b}:=X_{b}=\beta x_{s}$. This yields after substitution

$$\frac{1}{x_{b}}\rho=v_{u}\frac{\frac{m_{e}}{d_{e}}\beta\gamma x_{s}^{2}}{\frac{m_{e}}{d_{e}}\beta\gamma x_{s}^{2}+k_{u}}=v_{u}\frac{m_{e}x_{s}^{2}}{m_{e}x_{s}^{2}+q}$$

where $q=k_{u}\frac{d_{e}}{\beta\gamma}$. Enzymes are released at rate $m_{e}$ and enzyme production was assumed to have additional respiration costs $m_{e}r_{e}$ so that the total enzyme cost is $m_{e}(1+r_{e})$. Lastly, a non-adaptable maintenance cost with specific rate $r_{b}$ was considered so that the net specific growth rate is obtained as given in Box 1 as

$$\mu=\underset{Adaptable benefit}{\underbrace{\overset{Growth rate}{\overbrace{yv_{u}\frac{m_{e}x_{s}^{2}}{m_{e}x_{s}^{2}+q}}}}}-\underset{Adaptable cost}{\underbrace{\overset{Emzyme production}{\overbrace{m_{e}\left( 1+r_{e} \right)}}}}-\underset{Non-adaptable}{\underbrace{\overset{\mathrm{Maintenance}}{\overbrace{r_{b}}}}}$$

where $y$ is the assimilation efficiency. In the notation of our framework the adaptable benefit is $B=yv_{u}\frac{m_{e}x_{s}^{2}}{m_{e}x_{s}^{2}+q}$, the adaptable cost is $C=m_{e}(1+r_{e})$, and the non-adaptable cost is $K^{'}=-r_{b}$. Only $B$ and $C$ are relevant to compute the fitness as $\frac{\partial K^{'}}{\partial m_{e}}=0$.

Abs *et al.* (2025) considered the same system as Calabrese *et al.* (2022) with state variables $\boldsymbol{x}=(x_{s},x_{d},x_{b},x_{e})$ defined as before. Abs *et al.* (2025) conceptually separated the microbial population in a resident population $x_{r}:=x_{b}$ and a mutant population $x_{m}$. The mutant was assumed to be rare ($x_{m}\ll x_{r}$) so it does not affect the resident dynamics during its initial growth phase. Microbes invest a fraction $0<\phi<1$ of the assimilation rate $y\rho$ into enzyme production and use the reminder $\left( 1-\phi\right)y\rho$ to produce new biomass. Abs *et al.* (2025) assumed that enzymes will stay close to the cell that released them and thus $x_{d}$ will become available primarily locally. This means that a microbe that invests more into enzymes “sees more” of the average DOC concentration $x_{d}$ than a microbe that produces less enzymes. As a result, the resident uptake rate $\rho_{r}(\phi_{r})$ can be described as

$$\frac{1}{x_{r}}\rho_{r}(\phi_{r})=v_{u}\frac{\left( 1+c\left( \phi_{r}-\phi_{r} \right) \right)x_{d}}{\left( 1+c\left( \phi_{r}-\phi_{r} \right) \right)x_{d}+k_{u}}=v_{u}\frac{x_{d}}{x_{d}+k_{u}}$$

where $c$ is a function that quantifies the relative advantage of producing more enzymes. Since the resident population was assumed to be homogeneous, no organism has an advantage over another and $c\left( \phi_{r}-\phi_{r} \right)=0$. In contrast, a mutant was defined as having a different enzyme allocation trait $\phi_{m}$, so the mutant uptake rate is

$$\frac{1}{x_{m}}\rho_{m}(\phi_{m},\phi_{r})=v_{u}\frac{\left( 1+c\left( \phi_{m}-\phi_{r} \right) \right)x_{d}}{\left( 1+c\left( \phi_{m}-\phi_{r} \right) \right)x_{d}+k_{u}}$$

where the function $c$ is defined so that $c\left( \phi_{m}-\phi_{r} \right)>0$ for $\phi_{m}>\phi_{r}$ and $c\left( \phi_{m}-\phi_{r} \right)<0$ for $\phi_{m}<\phi_{r}$. The associated enzyme production costs are then given by $\phi_{r}y\rho_{r}(\phi_{r})$ and $\phi_{m}y\rho_{r}(\phi_{m},\phi_{r})$ for the resident and mutant respectively.

Within the framework of adaptive dynamics, Abs *et al.* (2025) additionally assumed that the environment is at an ecological equilibrium with respect to the resident, that is $\boldsymbol{x}:=\boldsymbol{X}\left. \right|_{\phi_{r}}=f(\boldsymbol{p},\phi_{r})$ (where $\boldsymbol{X}\left. \right|_{\phi_{r}}$ are the solutions to $\frac{d\boldsymbol{x}}{dt}\left. \right|_{\phi_{r}}=0$ given the resident trait $\phi_{r}$). The mutant uptake rate at the equilibrium state set by the resident is then

$$\frac{1}{x_{m}}\rho_{m}\left( \phi_{m},\phi_{r} \right)\left. \right|_{\boldsymbol{x=X}\left. \right|_{\phi_{r}}}=v_{u}\frac{\left( 1+c\left( \phi_{m}-\phi_{r} \right) \right)X_{d}\left. \right|_{\phi_{r}}}{\left( 1+c\left( \phi_{m}-\phi_{r} \right) \right)X_{d}\left. \right|_{\phi_{r}}+k_{u}}$$

Next, the invasion fitness $s(\phi_{m},\phi_{r})$ is found as the initial specific growth rate of the mutant while it is still rare and given as

$$s\left( \phi_{m},\phi_{r} \right)=\underset{Adaptable benefit}{\underbrace{\overset{Growth rate}{\overbrace{yv_{u}\frac{\left( 1+c\left( \phi_{m}-\phi_{r} \right) \right)X_{d}\left. \right|_{\phi_{r}}}{\left( 1+c\left( \phi_{m}-\phi_{r} \right) \right)X_{d}\left. \right|_{\phi_{r}}+k_{u}}}}}}-\underset{Adaptable cost}{\underbrace{\overset{Enzyme production}{\overbrace{\phi_{m} yv_{u}\frac{\left( 1+c\left( \phi_{m}-\phi_{r} \right) \right)X_{d}\left. \right|_{\phi_{r}}}{\left( 1+c\left( \phi_{m}-\phi_{r} \right) \right)X_{d}\left. \right|_{\phi_{r}}+k_{u}}}}}}-\underset{Non-adaptable}{\underbrace{\overset{\mathrm{Mortality}}{\overbrace{d}}}}$$

In the notation of our framework the adaptable benefit is $B=yv_{u}\frac{\left( 1+c\left( \phi_{m}-\phi_{r} \right) \right)X_{d}\left. \right|_{\phi_{r}}}{\left( 1+c\left( \phi_{m}-\phi_{r} \right) \right)X_{d}\left. \right|_{\phi_{r}}+k_{u}}$, the adaptable cost is $C=\phi_{m} yv_{u}\frac{\left( 1+c\left( \phi_{m}-\phi_{r} \right) \right)X_{d}\left. \right|_{\phi_{r}}}{\left( 1+c\left( \phi_{m}-\phi_{r} \right) \right)X_{d}\left. \right|_{\phi_{r}}+k_{u}}$ and the non-adaptable cost is $K=-d$. Only $B$ and $C$ are relevant to compute the fitness as $\frac{\partial K}{\partial\phi_{m}}=0$.

As Abs *et al.* (2025) did not define the exact form of $c(\phi_{m},\phi_{r})$ beyond the conditions mentioned before, also $\frac{\partial c\left( \phi_{m},\phi_{r} \right)}{\partial\phi_{m}}$ and higher derivatives remain undefined so that the necessary conditions for a continuously stable strategy (namely, $\frac{\partial^{2}s\left( \phi_{m},\phi_{r} \right)}{\partial\phi_{m}^{2}}<0$ and $\frac{\partial^{2}s\left( \phi_{m},\phi_{r} \right)}{\partial\phi_{m}^{2}}+\frac{\partial^{2}s\left( \phi_{m},\phi_{r} \right)}{\partial\phi_{m}\partial\phi_{r}}<0$ at the optimal trait $\phi^{*}=\phi_{m}=\phi_{r}$) cannot be evaluated. Abs *et al.* (2025) assumed without loss of generality, that $c(\phi_{m},\phi_{r})$ satisfies these conditions.

*Example presented in Box 2:*

Manzoni *et al.* (2023) focus on the decomposition of a given amount of substrate by microbial consumption without explicitly describing enzyme production and microbial and DOC dynamics. Microbes consume and thereby decompose substrate at a rate $\rho(t)$ that was not further specified but is expected to emerge as a result of the EEO approach. Biomass was assumed to be in quasi-steady state so that growth $g$ is equivalent to microbial decay. Substrate dynamics were described by

$$\frac{dx_{s}}{dt}=-\overset{\begin{aligned} \mathrm{Microbial} \\ \mathrm{consumption} \end{aligned}}{\overbrace{\rho\left( t \right)}}-\overset{\begin{aligned} \mathrm{Abiotic} \\ \mathrm{loss} \end{aligned}}{\overbrace{\mathcal{l}_{s}x_{s}}}+\overset{\begin{aligned} Recycling of \\ \mathrm{necromass} \end{aligned}}{\overbrace{r_{n}g}}$$

where $\mathcal{l}_{s}$ is a linear loss rate coefficient and $r_{n}$ the fraction of necromass that is recycled as substrate. Manzoni *et al.* (2023) assumed microbial growth to be a saturating function of the consumption rate $\rho(t)$ and microbial maintenance respiration $r_{b}$ was subtracted from $\rho$ (so that growth cannot occur if $\rho<r_{b}$)

$$g\left( \rho\left( t \right) \right)=yk_{u}^{'}\frac{\rho\left( t \right)-r_{b}}{\rho\left( t \right)+k_{u}^{'}}=\underset{Adaptable benefit}{\underbrace{\overset{Growth rate}{\overbrace{\rho\left( t \right)\frac{yk_{u}^{'}}{\rho\left( t \right)+k_{u}^{'}}}}}}-\underset{Adaptable cost}{\underbrace{\overset{\mathrm{Maintenance}}{\overbrace{r_{b}\frac{yk_{u}^{'}}{\rho\left( t \right)+k_{u}^{'}}}}}}$$

In this optimal control approach microbes were faced with the decision when to consume substrate and at what rate. Quick consumption would make growth less efficient but diminish relative maintenance costs – slow consumption in turn leaves more of the substrate to be depleted by competing processes ($\mathcal{l}_{s}x_{s}$). In the simplest case analyzed by Manzoni *et al.* (2023), it was assumed that $r_{b}=r_{n}=0$. As $r_{b}=0$, in the notation of our framework also the adaptable costs $C=0$. Despite these simplifications, optimization of $\rho$ is possible as the adaptable benefits remain ($B=\rho\left( t \right)\frac{yk_{u}^{'}}{\rho\left( t \right)+k_{u}^{'}}$) – and due to the competition for substrate ($\mathcal{l}_{s}x_{s}$) there remains an “opportunity cost” of not consuming.

Manzoni *et al.* (2023) obtained the optimal solution $\rho^{*}(t)$ (or expressed in the state variable space as $\rho^{*}(x_{s})$) that maximizes $g$ over the time interval from $t=0$ to the final time $T$ using the Pontryagin Maximum Principle as

$$\rho^{*}\left( x_{s} \right)=\sqrt{\mathcal{l}_{s}k_{u}^{'}x_{s}}$$

The final decomposition time $T$ is also found from the optimization, as this was set up as a “free terminal time problem”.

The solution of this problem represents the optimal compromise between reduced growth efficiency at high consumption rates (growth saturates at increasing $\rho$) and abiotic losses at low consumption rates (less of the substrate can be used). In the initial phase of decomposition, optimal consumption should be high to avoid large losses because of abiotic losses. As less substrate remains, abiotic losses become smaller and consumption can slow down in order to become more efficient. Manzoni *et al.* (2023) could recast the solution in the time domain (i.e., $\rho$ as a function of $t$) or in the state variable domain ($\rho$ as a function of $x_{s}$).

A pedagogic presentation of this model, including scripts for use in mathematics classes, is provided in a subsequent work (Ledder & Manzoni 2024).

## **Additional Details on Toy Model**

The full description of our toy model is presented in Box 3 of the main text. Here we provide parameter values used for the exemplary simulations (Table S2), analytic solutions for all EEO approaches, and a more detailed derivation of the adaptive dynamics approach.

Table S2 Parameter values used in toy model simulations. If not indicated differenetly, parameter values are taken from ranges and “baseline” parameter values in Schwarz et al. (2024), who complied parameter values from Cotrufo & Lavallee (2022), Hararuk et al. (2015) and Tao et al. (2023).

| **Symbol** | **Definition** | **Unit** | **Value** | **Note** |
| --- | --- | --- | --- | --- |
| $c_{0}$ | Competition asymmetry factor | d | $4 c_{0}^{min}$ | Assumed |
| $d_{e}$ | Enzyme decay rate coefficient | d^-1^ | $2.49\times{10}^{-2}$ |  |
| $I$ | External SOC input rate | mg C g^-1^ d^-1^ | $1.88\times{10}^{-4}- 2.43\times{10}^{1}$ |  |
| $k_{u}$ | Half-saturation constant | mg C g^-1^ | $3.00\times{10}^{2}$ |  |
| $\mathcal{l}_{s}$ | Non-microbial SOC loss rate coefficient | d^-1^ | $1.00\times{10}^{-4}$ | Assumed |
| $m_{e}$ | Enzyme production rate coefficient | d^-1^ | optimized or $3.78\times{10}^{-3}$ | Optimized. In “fix” scenario set to mean of all optimized values. |
| $v_{u}$ | Maximum breakdown rate coefficient | d^-1^ | $5.96\times{10}^{1}$ |  |
| $x_{s}$ | Set SOC content in instantaneous optimization | mg C g^-1^ | ${1.00\times10}^{-1}-{2.00\times10}^{2}$ | Assumed |
| $y$ | Microbial substrate assimilation efficiency | 1 | $0.30$ | Assumed |
| $\alpha$ | Fraction of accessible reactive sites of SOC | 1 | $1.00\times{10}^{-3}$ | Assumed |
| $\beta$ | Proportionality factor of microbial biomass to SOC | 1 | $0.05$ | Assumed |
| $\varepsilon_{e}$ | Enzyme cost | 1 | $1.00\times{10}^{1}$ | Assumed |

*Analytic solution of EEO approaches*

For **instantaneous maximization** of the net specific growth rate, we find analytically the microbial enzyme production rate $m_{e}^{inst*}$ that maximizes microbial growth by solving $\frac{\partial\mu}{\partial m_{e}}=0$ for $m_{e}$ and checking that $\frac{\partial^{2}\mu}{\partial m_{e}^{2}}\left. \right|_{m_{e}^{inst*}}<0$ as

$$m_{e}^{inst*}=\left( \frac{k_{u}}{x_{s}}+1 \right)\left[ \left( \frac{v_{u}y}{d_{e}\varepsilon_{e}}\frac{x_{s}}{k_{u}+x_{s}} \right)^{\frac{1}{2}}-1 \right]\frac{d_{e}\alpha}{\beta}$$

Physically meaningful solutions are obtained for $x_{s}>k_{u}\left( \frac{v_{u}y}{d_{e}\varepsilon_{e}}-1 \right)^{-1}$. As $x_{s}$ increases, the square root term on the right-hand side (RHS) saturates ($\frac{x_{s}}{k_{u}+x_{s}}\to1$ for $k_{u}\ll x_{s}$) and $m_{e}^{inst*}(x_{s})\propto\frac{1}{x_{s}}$. As $x_{s}\to\infty$ eventually $\frac{k_{u}}{x_{s}}\to0$ and thus $\lim_{x_{s}\to\infty} m_{e}^{inst*}(x_{s})=\left[ \left( \frac{v_{u}y}{d_{e}\varepsilon_{e}} \right)^{\frac{1}{2}}-1 \right]\frac{d_{e}\alpha}{\beta}$ approaches a constant value.

In turn, as $x_{s}$ decreases and $x_{s}\to k_{u}\left( \frac{v_{u}y}{d_{e}\varepsilon_{e}}-1 \right)^{-1}$ the RHS term in square brackets becomes very small and dominates $m_{e}^{inst*}(x_{s})$. This gives rise to the observed patter in Figure 4a.

For **equilibrium maximization** of the net specific growth rate, we find analytically the microbial enzyme production rate $m_{e}^{eq*}$ that maximizes microbial growth by solving $\frac{\partial\mu\left. \right|_{\boldsymbol{x=X}}}{\partial m_{e}}=0$ for $m_{e}$ and checking that $\frac{\partial^{2}\mu\left. \right|_{\boldsymbol{x=X}}}{\partial m_{e}^{2}}\left. \right|_{m_{e}^{eq*}}<0$ as

$$m_{e}^{eq*}=\frac{d_{e}\alpha}{\beta}\frac{k_{u}}{I}\left[ \left( \Delta+d_{e}\alpha\varepsilon_{e} \right)\left( \frac{v_{u}}{d_{e}\varepsilon_{e}}\frac{\Pi}{\Delta} \right)^{\frac{1}{2}}-\left( \Pi+\alpha v_{u} \right) \right]$$

with $\Delta=y\frac{I}{k_{u}}+d_{e}\alpha\varepsilon_{e}$ and $\Pi=\frac{I}{k_{u}}+\mathcal{l}_{s}+\alpha v_{u}$.

As $m_{e}^{eq*}$ is not a direct function of $x_{s}$ it is not possible to derive a direct intuition for how $m_{e}^{eq*}$ and SOC content co-vary. At high input rates ($I\to\infty$ , which eventually leads to high SOC, Figure 4b), $\frac{k_{u}}{I}\to0$ and thus $\Delta=\frac{I}{k_{u}}\left( y+\frac{k_{u}}{I}d_{e}\alpha\varepsilon_{e} \right)\approx\frac{I}{k_{u}}y$; $\Pi=\frac{I}{k_{u}}\left( 1+\frac{k_{u}}{I}\left( \mathcal{l}_{s}+\alpha v_{u} \right) \right)\approx\frac{I}{k_{u}}$ and thus

$$\lim_{I\to\infty} m_{e}^{eq*}(I)\approx\lim_{I\to\infty}\frac{d_{e}\alpha}{\beta}\left[ \left( y+\frac{k_{u}}{I}d_{e}\alpha\varepsilon_{e} \right)\left( \frac{v_{u}}{d_{e}\varepsilon_{e}y} \right)^{\frac{1}{2}}-\left( 1+\frac{k_{u}}{I}\alpha v_{u} \right) \right]=\frac{d_{e}\alpha}{\beta}\left[ \left( \frac{v_{u}y}{d_{e}\varepsilon_{e}} \right)^{\frac{1}{2}}-1 \right]$$

which is equivalent to $\lim_{x_{s}\to\infty} m_{e}^{inst*}(x_{s})$.

With **adaptive dynamics**, we find analytically the microbial enzyme production rate $m_{e}^{ad*}$ that is a continuous stable strategy as

$$m_{e}^{ad*}=\frac{d_{e}\alpha\varepsilon_{e}}{\alpha v_{u}yc_{0}-\beta\varepsilon_{e}}\left( \frac{k_{u}}{I}\left( \frac{\beta\varepsilon_{e}}{yc_{0}}+\mathcal{l}_{s} \right)+1 \right)$$

where $c_{0}=\frac{\partial c\left( m_{e}^{r},m_{e}^{m} \right)}{\partial m_{e}^{m}}\left. \right|_{m_{e}^{r}=m_{e}^{m}=m_{e}^{ad*}}$ is a new unknown parameter that quantifies how much producing more enzymes increases the access to depolymerization products relative to the resident. For $m_{e}^{ad*}$ to always be positive it is required that $c_{0}>\frac{\beta\varepsilon_{e}}{\alpha v_{u}y}=c_{0}^{min}$. For $m_{e}^{ad*}$ to be a continuous stable strategy it is required that $c_{0}$ is such that $\frac{\partial^{2}c\left( m_{e}^{r},m_{e}^{m} \right)}{\partial{m_{e}^{m}}^{2}}\left. \right|_{m_{e}^{r}=m_{e}^{m}=m_{e}^{ad*}}<0$ and $\frac{\partial^{2}c\left( m_{e}^{r},m_{e}^{m} \right)}{\partial{m_{e}^{m}}^{2}}\left. \right|_{m_{e}^{r}=m_{e}^{m}=m_{e}^{ad*}}+\frac{\partial^{2}c\left( m_{e}^{r},m_{e}^{m} \right)}{\partial m_{e}^{m}\partial m_{e}^{r}}\left. \right|_{m_{e}^{r}=m_{e}^{m}=m_{e}^{ad*}}<0$ (compare the explanation on the example of Abs *et al.* (2025) and Box 1).

*Details on the adaptive dynamics approach*

The growth dynamic of the mutant is found from the generalized resident growth dynamic at equilibrium (eq. 5 in Box 3)

$$\left( \frac{1}{x_{b}}\frac{dx_{b}}{dt} \right)_{\boldsymbol{x}=\boldsymbol{X}\left. \right|_{m_{e}^{r}}}=y\rho\left( \boldsymbol{p},\boldsymbol{X}\left. \right|_{m_{e}^{r}},m_{e}^{r} \right)c\left( m_{e}^{r},m_{e}^{r} \right)-\varepsilon_{e}m_{e}^{r}-d(\boldsymbol{p},\boldsymbol{X}\left. \right|_{m_{e}^{r}} ,m_{e}^{r})=0$$

with the SOC degradation rate given by

$$\rho\left( \boldsymbol{p},\boldsymbol{X}\left. \right|_{m_{e}^{r}},m_{e}^{r} \right)=v_{u}\frac{\alpha X_{s}\left. \right|_{m_{e}^{r}} \frac{m_{e}^{r}}{d_{e}}}{\alpha k_{u}+\alpha X_{s}\left. \right|_{m_{e}^{r}}+\frac{m_{e}^{r}}{d_{e}}\beta X_{s}\left. \right|_{m_{e}^{r}}}$$

and the decay function $d(\boldsymbol{p},\boldsymbol{X}\left. \right|_{m_{e}^{r}} ,m_{e}^{r})$ so that $\frac{1}{x_{b}}\frac{dx_{b}}{dt}=0$. We assume that both these rates, the flux at which degradation products become available $\rho\left( \boldsymbol{p},\boldsymbol{X}\left. \right|_{m_{e}^{r}},m_{e}^{r} \right)$ and the rate at which microbes decay $d\left( \boldsymbol{p},\boldsymbol{X}\left. \right|_{m_{e}^{r}},m_{e}^{r} \right)$ (e.g., because of density control) are dominated by the resident and the mutant’s influence on them is negligible as $x_{m}\ll x_{b}$. For instance, for the precise degradation rate $\rho$ (eq. (3) in Box 3) in presence of the mutant and resident would be

$$\rho=v_{u}\frac{\alpha x_{s} (x_{e}^{r}+x_{e}^{m})}{\alpha k_{u}+\alpha x_{s}+(x_{e}^{r}+x_{e}^{m})}$$

where $x_{e}^{r}$ and $x_{e}^{m}$ are the enzyme concentrations resulting from resident, respectively mutant production. Yet, since $x_{m}\ll x_{r}$ also $x_{e}^{m}\ll x_{e}^{r}$ and thus we can approximate $x_{e}^{r}+x_{e}^{m}\approx x_{e}^{r}$.

The mutant’s strategy thus affects its fitness only by modulating its relative access to degradation products (the adaptable benefit), which is captured by $c\left( m_{e}^{m},m_{e}^{r} \right)$, and the investment into enzyme production $\varepsilon_{e}m_{e}^{m}$ (the adaptable cost). The mutant growth dynamic is then given by

$$\left( \frac{1}{x_{m}}\frac{dx_{m}}{dt} \right)_{\boldsymbol{x}=\boldsymbol{X}\left. \right|_{m_{e}^{r}}}=y\rho\left( \boldsymbol{p},\boldsymbol{X}\left. \right|_{m_{e}^{r}},m_{e}^{r} \right)c\left( m_{e}^{m},m_{e}^{r} \right)-\varepsilon_{e}m_{e}^{m}-d(\boldsymbol{p},\boldsymbol{X}\left. \right|_{m_{e}^{r}} ,m_{e}^{r})$$

which is equivalent to the invasion fitness $s\left( m_{e}^{r},m_{e}^{m} \right)$ (eq. 6 in Box 3).

**References**

Abs, E., Leman, H. & Ferriere, R. (2020). A multi-scale eco-evolutionary model of cooperation reveals how microbial adaptation influences soil decomposition. *Commun. Biol.*, 3, 520.

Abs, E., Saleska, S.R., Allison, S.D., Ciais, P., Song, Y., Weintraub, M.N., *et al.* (2025). Microbiome Adaptation Could Amplify Modeled Projections of Global Soil Carbon Loss With Climate Warming. *Glob. Change Biol.*, 31, e70301.

Allison, S.D. (2014). Modeling adaptation of carbon use efficiency in microbial communities. *Front. Microbiol.*, 5, 1–9.

Averill, C. (2014). Divergence in plant and microbial allocation strategies explains continental patterns in microbial allocation and biogeochemical fluxes. *Ecol. Lett.*, 17, 1202–1210.

Bonner, M.TL., Franklin, O., Hasegawa, S. & Näsholm, T. (2022). Those who can don’t want to, and those who want to can’t: An eco-evolutionary mechanism of soil carbon persistence. *Soil Biol. Biochem.*, 174, 108813.

Calabrese, S., Mohanty, B.P. & Malik, A.A. (2022). Soil microorganisms regulate extracellular enzyme production to maximize their growth rate. *Biogeochemistry*, 158, 303–312.

Chakrawal, A., Lindahl, B.D. & Manzoni, S. (2024). Modelling optimal ligninolytic activity during plant litter decomposition. *New Phytol.*, 243, 866–880.

Cotrufo, M.F. & Lavallee, J.M. (2022). Soil organic matter formation, persistence, and functioning: A synthesis of current understanding to inform its conservation and regeneration. In: *Advances in Agronomy*, 172 (ed. Sparks, D.L.). Academic Press, pp. 1–66.

Franklin, O., Hall, E.K., Kaiser, C., Battin, T.J. & Richter, A. (2011). Optimization of Biomass Composition Explains Microbial Growth-Stoichiometry Relationships. *Am. Nat.*, 177, E29–E42.

Hararuk, O., Smith, M.J. & Luo, Y. (2015). Microbial models with data‐driven parameters predict stronger soil carbon responses to climate change. *Glob. Change Biol.*, 21, 2439–2453.

Heaton, L.L.M., Jones, N.S. & Fricker, M.D. (2016). Energetic Constraints on Fungal Growth. *Am. Nat.*, 187, E27–E40.

Ledder, G. & Manzoni, S. (2024). An optimal control problem for resource utilisation by microorganisms. *Int. J. Math. Educ. Sci. Technol.*, 55, 547–564.

Manzoni, S., Čapek, P., Mooshammer, M., Lindahl, B.D., Richter, A. & Šantrůčková, H. (2017). Optimal metabolic regulation along resource stoichiometry gradients. *Ecol. Lett.*, 20, 1182–1191.

Manzoni, S., Chakrawal, A. & Ledder, G. (2023). Decomposition rate as an emergent property of optimal microbial foraging. *Front. Ecol. Evol.*, 11.

Parnas, H. & Cohen, D. (1976). Optimal strategy for metabolism of reserve materials in microorganisms. *J. Theor. Biol.*, 56, 19–55.

Pfeiffer, T. & Bonhoeffer, S. (2002). Evolutionary Consequences of Tradeoffs between Yield and Rate of ATP Production. *Z. Für Phys. Chem.*, 216, 51–63.

Schwarz, E., Ghersheen, S., Belyazid, S. & Manzoni, S. (2024). When and why microbial-explicit soil organic carbon models can be unstable. *Biogeosciences*, 21, 3441–3461.

Sinsabaugh, R.L. & Moorhead, D.L. (1994). Resource allocation to extracellular enzyme production: A model for nitrogen and phosphorus control of litter decomposition. *Soil Biol. Biochem.*, 26, 1305–1311.

Smith, G.R. & Wan, J. (2019). Resource‐ratio theory predicts mycorrhizal control of litter decomposition. *New Phytol.*, 223, 1595–1606.

Tao, F., Huang, Y., Hungate, B.A., Manzoni, S., Frey, S.D., Schmidt, M.W.I., *et al.* (2023). Microbial carbon use efficiency promotes global soil carbon storage. *Nature*, 618, 981–985.

Vallino, J.J. (2010). Ecosystem biogeochemistry considered as a distributed metabolic network ordered by maximum entropy production. *Philos. Trans. R. Soc. B-Biol. Sci.*, 365, 1417–1427.

Vetter, Y.A., Deming, J.W., Jumars, P.A. & Krieger-Brockett, B.B. (1998). A predictive model of bacterial foraging by means of freely released extracellular enzymes. *Microb. Ecol.*, 36, 75–92.

Weverka, J.R., Moeller, H.V. & Schimel, J.P. (2023). Chemodiversity controls microbial assimilation of soil organic carbon: A theoretical model. *Soil Biol. Biochem.*, 187, 109161.

Wutzler, T., Reimers, C., Ahrens, B. & Schrumpf, M. (2024). Optimal enzyme allocation leads to the constrained enzyme hypothesis: the Soil Enzyme Steady Allocation Model (SESAM; v3.1). *Geosci. Model Dev.*, 17, 2705–2725.

Wutzler, T., Zaehle, S., Schrumpf, M., Ahrens, B. & Reichstein, M. (2017). Adaptation of microbial resource allocation affects modelled long term soil organic matter and nutrient cycling. *Soil Biol. Biochem.*, 115, 322–336.
